# Cycloastragenol Inhibits Colorectal Cancer Cell Metastasis via Epithelial–Mesenchymal Transition and the PI3K Signalling Pathway

**DOI:** 10.1111/jcmm.71128

**Published:** 2026-04-16

**Authors:** JingRong Miao, PanFeng Feng

**Affiliations:** ^1^ Department of Procurement Management Nantong First People's Hospital Nantong Jiangsu Province China; ^2^ Department of Pharmacy Nantong First People's Hospital Nantong Jiangsu Province China; ^3^ Nantong Key Laboratory of Innovative Research on Rheumatology and Immunology Nantong Jiangsu Province China; ^4^ Nantong Clinical Medical College of Kangda College of Nanjing Medical University Lianyungang Jiangsu Province China

**Keywords:** colorectal cancer, cycloastragenol, epithelial‐to‐mesenchymal transition, molecular docking, PI3K‐AKT signalling axis

## Abstract

Metastasis remains a critical factor contributing to the low survival rates in colorectal cancer. The pathways through which cycloastragenol suppresses colorectal cancer metastasis are poorly defined. This research directly investigates its anti‐metastatic mechanisms. The anti‐proliferative effects of cycloastragenol were assessed using CCK‐8 assays, while its impact on migration, invasion and apoptosis was evaluated via Transwell assays and flow cytometry. Analysis of protein expression was performed using Western blotting and immunofluorescence. Network pharmacology and molecular docking were employed to predict potential signalling pathways and binding interactions. Additionally, the establishment of an in vivo xenograft mice model enabled us to further validate the antitumour efficacy and mechanistic role of cycloastragenol. Cycloastragenol exhibited a dose‐ and time‐dependent suppression of colorectal cancer cell proliferation. Meanwhile, it inhibits proliferation and migration and promotes apoptosis in a concentration‐dependent manner. Cycloastragenol suppresses the activation of the EMT process. Based on an integrated network pharmacology and molecular docking approach, the PI3K/Akt signalling axis emerged as the foremost candidate mechanism. Cycloastragenol exhibits strong binding affinities with PI3K (interaction sites: ARG4, LYS720) and AKT (interaction sites: TYR18, LEU295), with binding energies of −9.0 kcal/mol and −9.3 kcal/mol, respectively. A marked suppression of tumour growth and metastasis was observed in xenograft models following cycloastragenol treatment. The antitumour efficacy of cycloastragenol is mediated primarily through the suppression of the PI3K/AKT signalling pathway, thereby suppressing EMT and impeding CRC progression. These findings provide a preclinical foundation for the potential clinical application of cycloastragenol in CRC therapy.

## 1 Introduction

Colorectal cancer (CRC) remains a global health challenge, imposing a significant burden due to its rising incidence and mortality [1]. Current projections indicate an alarming 60% rise in the global CRC burden by 2030 [2]. According to the 2020 Global Cancer Statistics Report, CRC is the third most frequently diagnosed malignancy, with 1.932 million new cases annually, and the second leading cause of cancer mortality, responsible for approximately 935,000 deaths. In China, the situation is particularly concerning, with CRC incidence ranking second (555,000 new cases) and mortality ranking fifth (286,000 deaths) [3]. Notably, recent epidemiological data reveal an age‐dependent escalation in both incidence and mortality rates within China [4]. A particularly alarming trend is the rising incidence of CRC, which has become the primary cause of cancer mortality in men under 50 and ranks second only to breast cancer in women within the same age group [5].

CRC is characterised by its pathological complexity, involving diverse genetic alterations and molecular mechanisms that affect both colon and rectal tissues [6]. Unfortunately, the disease is frequently diagnosed at intermediate or advanced stages. While chemotherapy remains a mainstay for improving patient outcomes and overall survival, its cytotoxic effects often lead to severe complications, including bone marrow suppression and life‐threatening adverse reactions [7]. Given these limitations and CRC's growing prevalence, there is an urgent need to refine existing clinical strategies and develop novel therapeutic agents [8]. Growing evidence has established the prognostic significance of pan‐immune‐inflammation value (PIV) and NP/LHb and Mono (NPM) model for assessing colorectal cancer progression [9, 10, 11]. Despite significant progress in CRC treatments, Traditional Chinese Medicine (TCM) remains a promising therapeutic approach owing to its unique multi‐target mechanisms, systemic regulation capacity and reduced adverse effects.

Among various bioactive compounds, cycloastragenol—a triterpene aglycone derived from Astragali Radix (Huangqi)—has garnered substantial research attention owing to its broad spectrum of pharmacological activities, such as anti‐ageing, antioxidant, anti‐inflammatory and bone metabolism‐modulating effects [12, 13]. Recent investigations have particularly focused on its anti‐tumour capabilities. Studies have demonstrated that cycloastragenol induces apoptosis and protective autophagy via the AMPK/ULK1/mTOR signalling axis in human non‐small cell lung cancer (NSCLC) cell lines [14]. Separately, cycloastragenol has been shown to inhibit constitutive STAT3 activation and enhance paclitaxel‐induced apoptosis in human gastric cancer cells [15]. Although one study confirmed cycloastragenol's ability to trigger apoptosis in CRC cells through p53 activation [16], its influence on colorectal cancer metastasis, especially regarding the modulation of epithelial–mesenchymal transition, has not been fully explored.

Epithelial–mesenchymal transition (EMT) is a fundamental process in which epithelial cells undergo a transformation to adopt a mesenchymal phenotype, granting them heightened migratory and invasive capabilities [17]. This phenomenon plays a pivotal role in tumour metastasis and contributes substantially to chemoresistance across multiple cancer types [18, 19, 20]. Consequently, EMT inhibition represents a promising therapeutic strategy to either prevent primary tumours from developing invasive properties or suppress recurrence following tumour resection [21].

In this study, we demonstrate that cycloastragenol effectively suppresses colorectal cancer cell metastasis and invasion by modulating the EMT process by targeting the PI3K‐AKT–mTOR signalling cascade. Complementary in vivo experiments further confirmed cycloastragenol's ability to inhibit tumour growth. These findings not only provide novel insights into cycloastragenol's anti‐CRC mechanisms but also position it as a promising candidate for development in both preventive and therapeutic contexts against colorectal cancer.

## 2 Materials and Methods

### 2.1 Chemicals, Reagents and Antibodies

Cycloastragenol was purchased from MedChemExpress (CAS: 78574‐94‐4). The protein's primary antibodies (Ki67, ab16667; PCNA, ab92552; Bcl‐2, ab182858; Bax, ab32503; cleaved caspase‐3, ab2302; caspase‐3, ab32351; E‐cadherin, ab314063; N‐cadherin, ab76011; Vimentin, ab20346; snail, ab216347; slug, ab314086; p‐PI3K, ab182651; PI3K, ab133595; p‐AKT, ab81283; AKT, ab8805; p‐mTOR, ab109268; mTOR, ab134903; GAPDH, ab8245) and secondary antibodies (Goat anti‐rabbit IgG H&L (HRP) (ab6721)) were purchased from Abcam Plc. 740 YP (CAS: 1236188‐16‐1) was purchased from Aladdin Industrial Corporation.

### 2.2 CCK8 Assay

HCT116 (SCSP‐5076), DLD‐1 (SCSP‐5241) and SW620 (SCSP‐5234) were purchased from National Collection of Authenticated Cell Cultures and cultured following the manufacturer's protocol. The STR authentication report for the cell line was provided and designated as ‘Certificate of STR Analysis of Cells’. Cells in the logarithmic growth phase (5 × 10^3^ cells/well) were seeded into 96‐well plates. After cell attachment, complete medium containing different concentrations of cycloastragenol (0, 25, 50 and 75 μM) was added. The cells were then cultured for 24, 48 and 72 h respectively in a cell culture incubator. Following incubation with CCK‐8 working solution for 1 h, cell viability was determined. Cell viability (%) = [(OD treatment group—OD blank well) / (OD drug group—OD blank well)] × 100%.

### 2.3 Western Blot Experiments

Cells were collected and washed with pre‐cold PBS. Total cellular protein was extracted using RIPA buffer supplemented with 1% protease inhibitor. Protein concentration was determined by the BCA method. Proteins (20 μg per lane) were separated by 8%–15% SDS‐PAGE and transferred onto PVDF membranes. The membranes were blocked for 1 h at room temperature (RT) with 5% non‐fat milk prepared in TBST. Subsequently, the membranes were incubated with primary antibodies (Ki67, 1/500; PCNA, ab92552, 1/50000; Bcl‐2, 1/2000; Bax, 1/2000; cleaved caspase‐3, 1/2000; caspase‐3, 1/5000; E‐cadherin, 1/1000; N‐cadherin, 1/1000; Vimentin, 1/1000; snail, 1/1000; slug, 1/1000; p‐PI3K, 1/500; PI3K, 1/1000; p‐AKT, 1/1000; AKT, 1/5000; p‐mTOR, 1/5000; mTOR, 1/10000; GAPDH, 1/1000) overnight at 4°C. After incubation, the membranes were washed with TBST and then incubated with corresponding secondary antibodies (Goat anti‐rabbit IgG H&L (HRP), 1: 2000) for 1 h at room temperature. The membranes were then incubated in exposure solution for approximately 1 min, followed by exposure for protein band detection. Protein band intensities were quantified using ImageJ software. The target protein levels were normalised to the average intensity of β‐actin from the same sample to obtain relative values. The average relative value of the control group was set as 1, and the relative protein levels of other groups were calculated by comparison to the control. The uncropped membrane were provided as Supporting Information of western blot.

### 2.4 Cell Apoptosis Assay

Single‐cell suspensions were prepared from cycloastragenol‐treated colorectal cancer cells in each experimental group. Following cell counting, aliquots containing 1 × 10^5^ cells were transferred to fresh centrifuge tubes and subjected to double staining using Annexin V‐FITC and propidium iodide (PI) in strict adherence to the kit instructions. Cell apoptosis rates were then quantified by flow cytometry (e.g., BD FACSCalibur) using the standard Annexin V‐FITC/PI detection method.

### 2.5 Transwell Experiment

Cell Migration Assay: Cells were resuspended at a density of 1.5 × 10^4^ cells per 200 μL. Then, 200 μL of serum‐free single‐cell suspension containing the corresponding drug concentration was added to the upper chamber of a 24‐well Transwell plate, while 600 μL of medium supplemented with 10% serum was added to the lower chamber. After 48 h of incubation, the chamber was fixed with methanol for 20 min and stained with crystal violet for 20 min. Excess dye was washed off with PBS, and the chamber was air‐dried before being photographed under a microscope.

Cell Invasion Assay: The invasion assay followed an identical procedure to the migration assay, with the exception that the Transwell inserts were pre‐coated with 50 μL of Matrigel matrix (diluted 1:8 in serum‐free medium) and allowed to gel for 5 h at 37°C prior to cell seeding. The number of cells that successfully invaded through the Matrigel and membrane barrier was quantified as described above.

### 2.6 Immunofluorescence Staining Analysis

Cells were fixed with 4% paraformaldehyde at room temperature for 10 min, followed by three washes with PBS (5 min each). The cells were then permeabilised with 0.25% Triton X‐100 at room temperature for 10 min and washed again three times with PBS (5 min each). After blocking with goat serum, the primary antibody was added, and the cells were incubated at 4°C for 24 h. Subsequently, the samples were thoroughly washed with PBS (three times, 5 min each) and then exposed to a fluorophore‐conjugated secondary antibody. Fluorescence signals representing the target protein distribution were visualised under a fluorescence microscope, and representative images were acquired for documentation.

### 2.7 Assessment of the Impact of Cycloastragenol on Tumour Proliferation In Vivo

All animal procedures were approved by the Animal Experiment Ethics Committee of Nantong University (Approval No. S20220919‐0152) and conducted in accordance with the ARRIVE guidelines. Animals were housed in a temperature‐ and humidity‐controlled SPF facility (25°C ± 2°C, 50% ± 5% humidity) under a 12 h light/dark cycle, with ad libitum access to food and water and randomly assigned to groups using a random number table method. The experiment was conducted in a single‐blind manner, as all assessed parameters were objective indicators. Sample size was estimated using degrees of freedom (E), where E = total number of animals − total number of groups. An E value between 10 and 20 indicates an appropriate animal sample size [22].

Subcutaneous Xenograft Model Establishment: To establish the subcutaneous tumour model, HCT116 cells (1 × 10^6^ cells/mice) were resuspended in PBS and inoculated bilaterally into the axillary regions of the mice (BALB/c, *n* = 5 per group). Once palpable tumours formed, their volumes were monitored every three days. Mice were randomly allocated into two groups when the average tumour volume reached approximately 100 mm^3^. The treatment group received cycloastragenol at doses of 100 or 200 mg/kg, whereas control animals received a corresponding volume of normal saline. Upon completion of the 14‐day dosing period, mice were deeply anaesthetised by intraperitoneal injection of ketamine (100 mg/kg) in combination with atropine (0.05 mg/kg, subcutaneous injection), and every effort was made to minimise distress. Subsequently, euthanasia was performed via cervical dislocation. Tumours were excised, weighed and measured. One portion of the tumour tissue was processed for immunohistochemical analysis, while another portion, along with organs such as the liver, was fixed for subsequent haematoxylin and eosin (H&E) staining.

Experimental Metastasis Model: A separate experimental metastasis model was generated by intravenously injecting HCT116 cells (1 × 10^6^ cells/mice) into the tail vein of nude mice (BALB/c). Two weeks post‐injection, the mice received cycloastragenol (200 mg/kg) as the therapeutic intervention. Following a 60‐day observation period, the mice were euthanised, and lung and other tissues were collected for analysis of metastatic foci. The methods of euthanasia in the tumour metastasis mice model were consistent with the subcutaneous tumour model. And then, the lungs/liver were photographed to quantify metastatic nodules. The study endpoint in the tumour metastasis mice model and subcutaneous tumour model was defined as when the tumour volume in any group exceeded the ethical limit of 1000 mm^3^. No animals died before meeting the criteria for euthanasia.

### 2.8 H&E Staining Experiments

After collection, specimens should be immediately fixed to preserve structural integrity. Perform graded dehydration using 80%, 90%, 95% and 100% ethanol sequentially, with each concentration applied for 2 h, followed by clearing with xylene. Infiltrate the tissue with paraffin wax to replace the clearing agent. Embedding: Place the wax‐infiltrated tissue in molten paraffin, allowing it to solidify completely around the specimen. Sectioning and floating: Use ophthalmic forceps to transfer sections onto a 40°C–45°C water bath, where surface tension and temperature help smooth out wrinkles. Mounting and baking: After complete flattening, transfer sections onto slides, drain excess water and bake at 60°C–65°C for 15–30 min to remove residual wax. The sections were processed through two changes of xylene (10 min each) for deparaffinisation, followed by rehydration in a series of descending ethanol concentrations (100% to 70%, 5 min each) before a final rinse in distilled water. Nuclei were stained with Harris haematoxylin (3–8 min), followed by washing in tap water, brief differentiation in 1% acid alcohol, bluing in 0.6% ammonia water and a final wash. Cytoplasm was counterstained with eosin (1–3 min). The sections were then dehydrated through an ascending ethanol series (95% to 100%), cleared in xylene (two changes, 5 min each), air‐dried and mounted with neutral balsam. Stained sections were examined and imaged under a microscope for subsequent analysis.

### 2.9 Network Pharmacology

Potential protein targets of cycloastragenol were identified using the PubChem and SwissTarget Prediction databases, yielding 109 unique targets after deduplication. Disease‐associated targets for colorectal cancer (CRC) were curated from the OMIM and GeneCards databases, resulting in 13,447 targets post‐consolidation. The overlapping targets between the compound and the disease were subsequently subjected to KEGG pathway enrichment analysis via the Metascape platform.

### 2.10 Molecular Docking

The X‐ray crystal structures of PI3K (PDB: 4JPS) and AKT (PDB: 7NH5) were acquired from the Protein Data Bank. All small molecule structures were protonated at pH 7.4 and converted to 3D format using Open Babel [23]. Protein and ligand preparation, including the addition of polar hydrogens and charge assignment, was performed with AutoDock Tools (ADT3). Docking grids were defined using AutoGrid, and molecular docking simulations were carried out with AutoDock Vina (v1.2.0) [24, 25]. The most favourable binding pose was selected for interaction analysis. For structural depiction in PyMOL, PI3K is shown as a slate cartoon, the ligand as a cyan stick model and key residues in the binding pocket as magenta sticks. Nonpolar hydrogens are omitted for clarity. Specific interactions—hydrogen bonds (yellow dashes), ionic bonds (magenta dashes) and hydrophobic contacts (green dashes)—are highlighted accordingly.

### 2.11 Statistical Analysis

Statistical analysis was performed using GraphPad Prism software (version 8). An unpaired *t*‐test was used for comparisons between two groups, and one‐way analysis of variance (ANOVA) was employed for comparisons among multiple groups. A *p*‐value of less than 0.05 was considered statistically significant.

## 3 Results

### 3.1 Cycloastragenol Inhibits the Proliferation of Colorectal Cancer Cells

**FIGURE 1:**
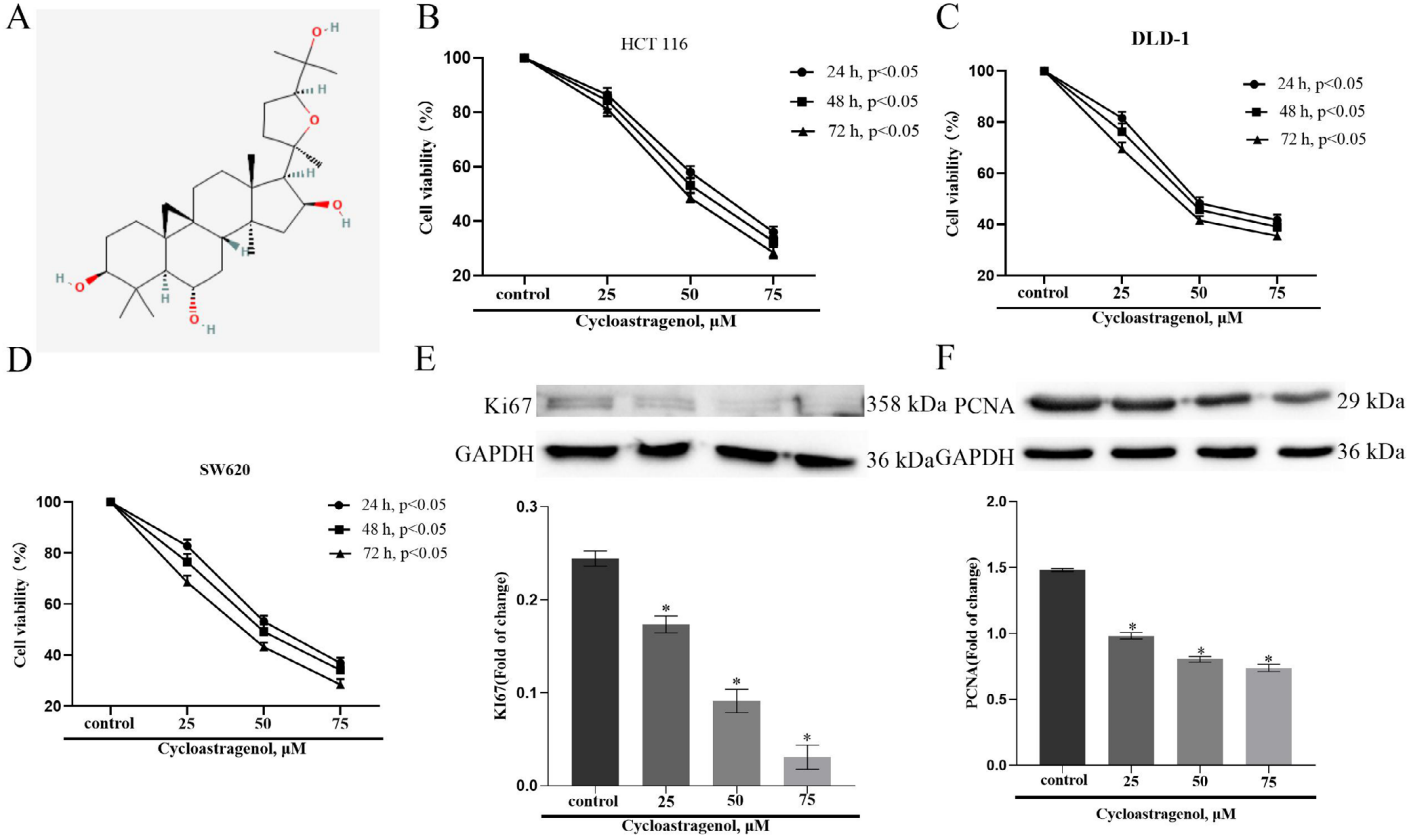
Effect of cycloastragenol on the proliferation of colorectal cancer cells was evaluated. (A) The chemical structure of cycloastragenol. (B–D) Viability was evaluated via CCK‐8 assay following treatment with a range of cycloastragenol doses (0, 25, 50, 75 μM) for 24 h, 48 h and 72 h in HCT116, DLD‐1 and SW620 cells. (E, F) Western blot detection of the effect of cycloastragenol on protein expression of Ki67 and PCNA in HCT116 cells. Data are presented as the mean ± SD. ^*^indicates the difference compared with the control group (*p* < 0.05), *n* = 3.

The effect of cycloastragenol on the proliferation of colorectal cancer cells was verified by the CCK8 assay. Figure 1A shows the chemical structure of cycloastragenol. The results indicated that, compared with the control group, cycloastragenol could inhibit the proliferation activity of colorectal cancer cells in a concentration‐dependent and time‐dependent manner (Figure 1B–D). Therefore, a 24‐h treatment duration was employed for subsequent experiments. PCNA and Ki67 are proliferation markers [26]. The results showed that cycloastragenol could inhibit the expression of PCNA and Ki67 proteins (Figure 1E,F).

### 3.2 Cycloastragenol Inhibits the Migration and Invasion of Colorectal Cancer Cells

**FIGURE 2:**
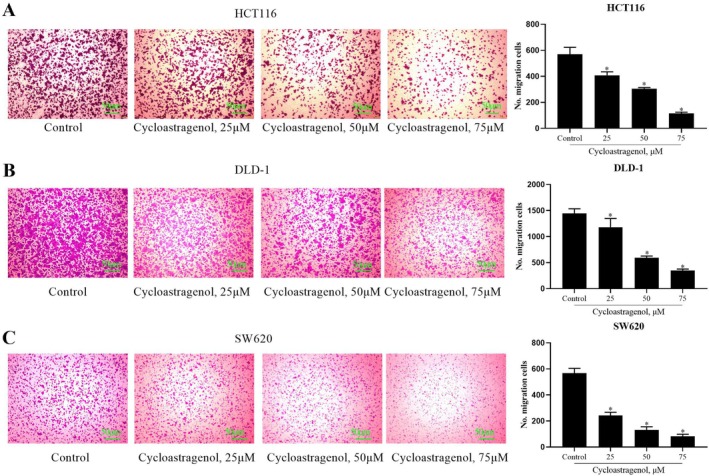
The effect of cycloastragenol on the migration of colorectal cancer cells was evaluated. (A–C) Migration ability was evaluated via transwell migration assay following treatment with a range of cycloastragenol doses (0, 25, 50, 75 μM) for 24 h in HCT116, DLD‐1 and SW620 cells. Data are presented as the mean ± SD. ^*^indicates the difference compared with the control group (*p* < 0.05), *n* = 3, Scale bar: 50 μm.

**FIGURE 3:**
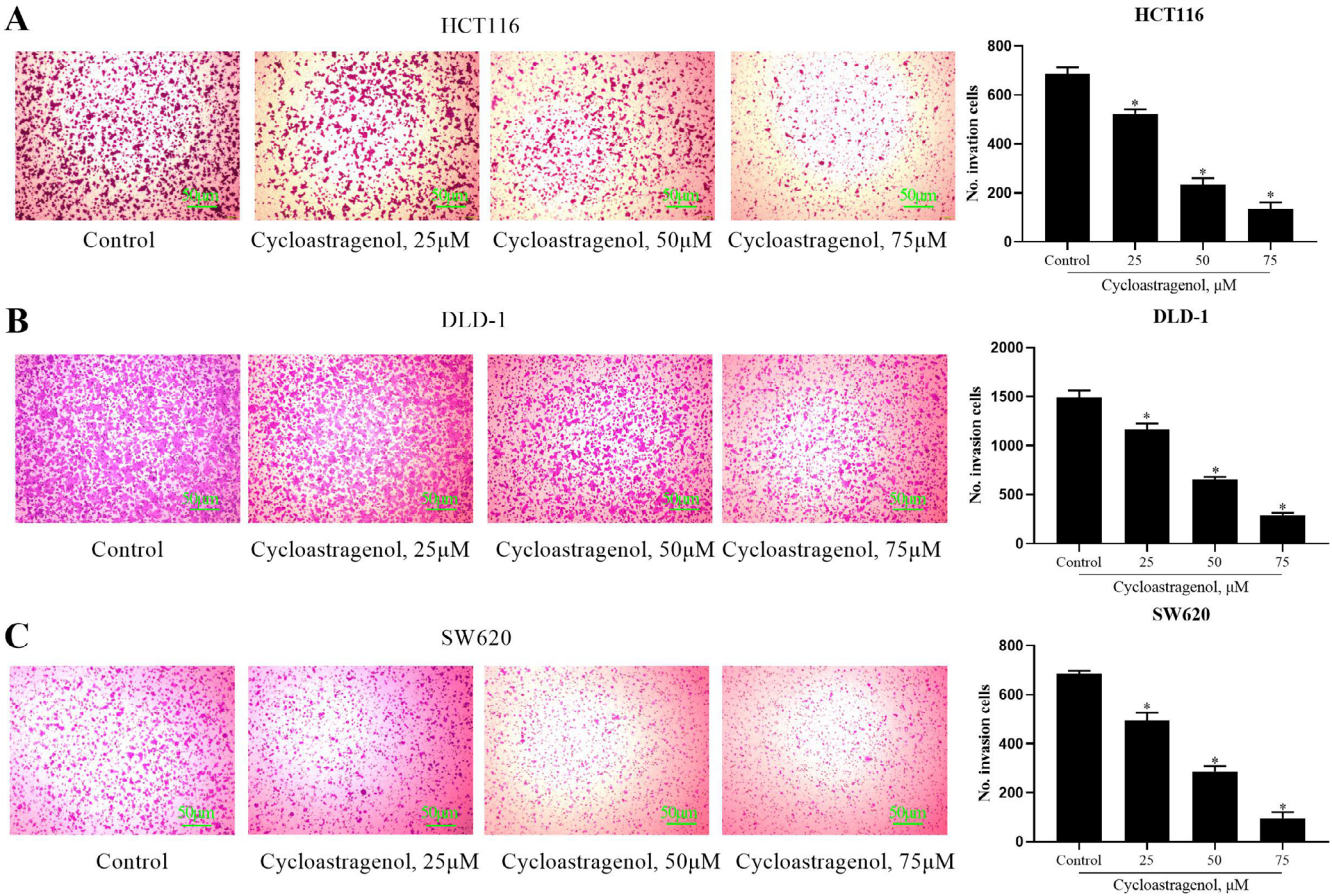
The effect of cycloastragenol on the invasion of colorectal cancer cells was evaluated. (A–C) Invasion ability was evaluated via transwell migration assay following treatment with a range of cycloastragenol doses (0, 25, 50, 75 μM) for 24 h in HCT116, DLD‐1 and SW620 cells. Data are presented as the mean ± SD. ^*^indicates the difference compared with the control group (*p* < 0.05), *n* = 3, Scale bar: 50 μm.

The results of the transwell migration assay demonstrated that, compared with the control group, cycloastragenol significantly inhibited the migration of colorectal cancer cells in a concentration‐dependent manner (Figure 2A–C). Similarly, the transwell invasion assay revealed that cycloastragenol exerted a concentration‐dependent inhibitory effect on the invasive capacity of colorectal cancer cells (Figure 3A–C).

### 3.3 Cycloastragenol Promotes Apoptosis in Colorectal Cancer Cells

**FIGURE 4:**
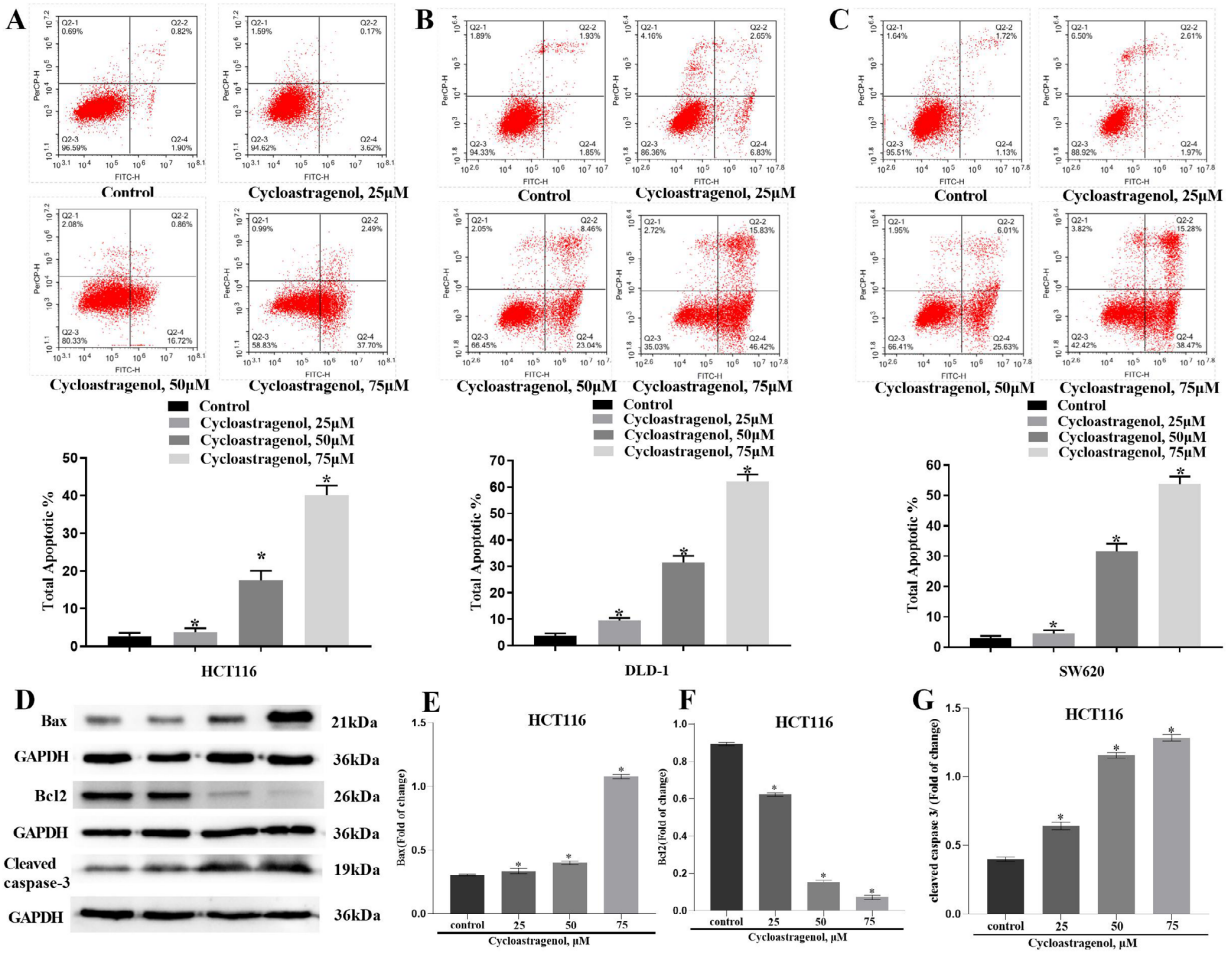
Cycloastragenol induced colon cancer cells apoptosis in a dose‐dependent manner. (A–C) HCT116, DLD‐1 and SW620 cells' apoptosis was detected by flow cytometry and quantified. (D–G) The expression level of Bax, Bcl‐2 and cleaved caspase‐3 proteins was assessed by western blot and quantified in HCT116 cells. Data are presented as the mean ± SD. ^*^indicates the difference compared with the control group (*p* < 0.05), *n* = 3.

Flow cytometry was used to detect the effect of cycloastragenol on apoptosis in colorectal cancer cells. Cycloastragenol exhibited a dose‐dependent capacity to significantly promote apoptosis in tumour cells (Figure 4A–C).

To delineate the mechanistic basis of cycloastragenol‐triggered apoptosis, we employed western blotting to quantify the expression of key apoptotic regulators, including the pro‐apoptotic protein BAX, the anti‐apoptotic protein Bcl‐2, and the executioner caspase, cleaved caspase‐3. As illustrated in Figure 4D–G, exposure of colorectal cancer cells to different concentrations of cycloastragenol led to a significant upregulation of BAX and cleaved caspase‐3 expression compared to the control group. Conversely, cycloastragenol treatment led to a decrease in Bcl‐2 expression in HCT116 cells.

### 3.4 Cycloastragenol Inhibits the Growth of HCT116‐Derived Tumours In Vivo

**FIGURE 5:**
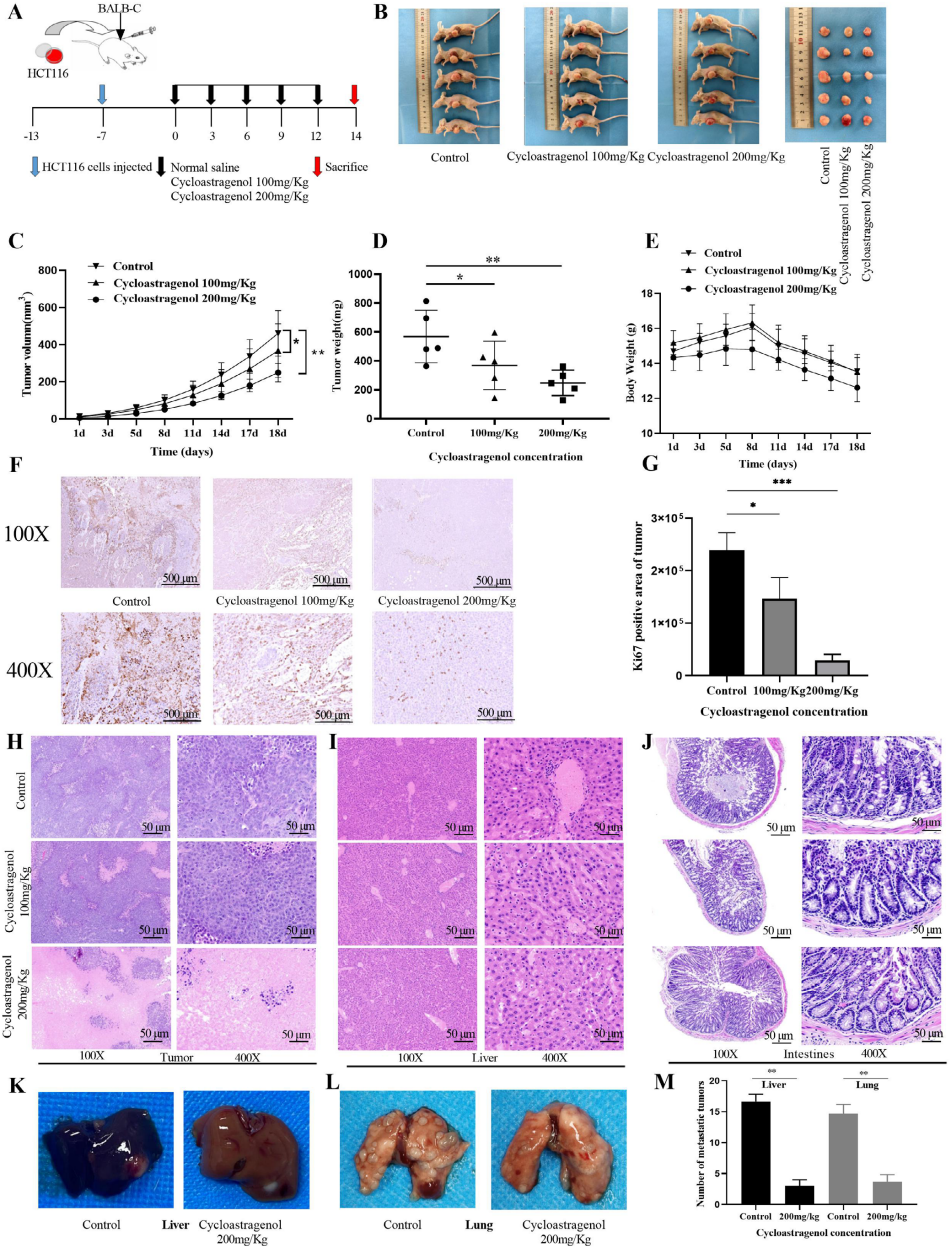
Inhibitory effects of cycloastragenol on the development and growth of xenograft colorectal tumours in vivo. (A) Flowchart of Animal Experiment. (B) Representative images of tumours in mice treated with cycloastragenol (100 mg/kg and 200 mg/kg). (C–E) Cycloastragenol reduced both the tumour volume and weight in mice, with no significant change in body weight. (F, G) Ki‐67 IHC staining of xenograft tumours. Original magnification 400×. Scale bar =500 μm. H‐scores of the Ki67 staining. (H–J) Representative images of tumour, liver and intestines with H&E staining, Scale bars = 50 μm. (K–M) Cycloastragenol inhibits liver and lung metastasis of colorectal tumours. *n* = 5. Data are presented as the mean ± SD. *p < 0.05, **p < 0.01 vs. the control group.

A mice model with tumours derived from HCT116 cells was created based on the schedule depicted in Figure 5A, in order to assess the in vivo inhibitory impact of cycloastragenol on colorectal cancer. Administration of cycloastragenol significantly suppressed the growth of HCT116‐derived tumours compared to the control group (Figure 5B), as evidenced by the tumour growth curves depicted in Figure 5C and the tumour weights shown in Figure 5D. Meanwhile, cycloastragenol had no significant effect on the weight changes of mice (Figure 5E). The immunohistochemistry (IHC) findings demonstrated that cycloastragenol administration resulted in differential down‐regulation of the expression levels of Ki67 (Figure 5F,G). The HE staining results revealed that in the control group, the tumour exhibited fullness and uniform distribution of tumour cells with disordered growth. With increasing doses of cycloastragenol (100 mg/kg, 200 mg/kg), extensive central necrosis and formation of vacuoles were observed within the tumour mass (Figure 5H). The HE staining results showed that cycloastragenol has no significant effect on the liver (Figure 5I). Furthermore, the intestinal crypt structures in the control group were disordered, with obvious morphological changes and extensive infiltration of inflammatory cells. The epithelial structure of the mucosa was damaged, and the thickness of the intestinal wall increased. In the high‐dose group (200 mg/kg), the crypt structures were basically normal, with only a small amount of inflammatory cells infiltrating. The epithelial structure of the mucosa was basically intact (Figure 5J). These findings suggest that the administration of cycloastragenol at doses of 100 mg·kg^−1^ and 200 mg·kg^−1^ was well‐tolerated in mice. To investigate the anti‐metastatic activity of cycloastragenol (200 mg·kg^−1^), we employed a murine tumour metastasis model. Results shown in Figure 5K–M revealed that cycloastragenol markedly suppressed liver and lung metastasis of colorectal tumours.

### 3.5 Cycloastragenol Inhibits the EMT Process of Colorectal Cancer Cells

**FIGURE 6:**
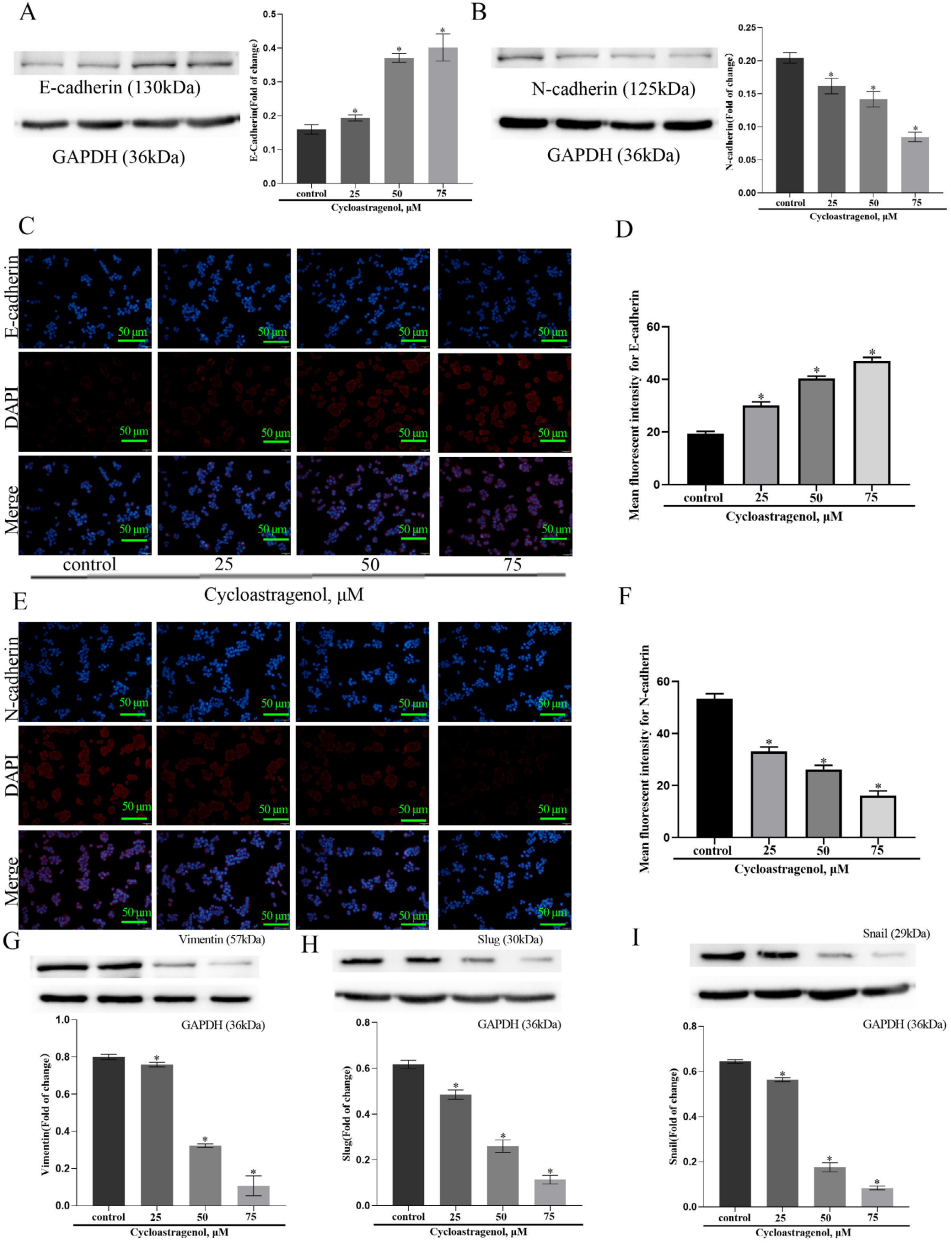
Cycloastragenol inhibits the EMT process of colorectal cancer cells. (A, B and G–I) Western blot was used to detect expression levels of E‐cadherin, N‐cadherin, vimentin, snail and slug. (C–F) Immunofluorescence analysis was used to detect expression levels of E‐cadherin and N‐cadherin. Data were expressed as mean ± SD, *n* = 3. Data are presented as the mean ± SD. *p < 0.05 vs. the control group, Scale bars = 50 μm.

During epithelial–mesenchymal transition (EMT), the loss of E‐cadherin and gain of N‐cadherin and Vimentin are hallmark molecular events [27], a process often driven by the overexpression of transcription factors like Slug and Snail [28]. Our western blot analysis demonstrated that cycloastragenol effectively reversed this signature by upregulating E‐cadherin (Figure 6A) and down‐regulating N‐cadherin (Figure 6B), Vimentin (Figure 6G), Snail (Figure 6H) and Slug (Figure 6I). Furthermore, cycloastragenol suppressed the mRNA expression of additional EMT‐related transcription factors, including ZEB1 and TWIST1, as shown in (Figure S1A,B). Convergent evidence from immunofluorescence confirmed the elevation of E‐cadherin (Figure 6C,D) and reduction of N‐cadherin (Figure 6E,F). The consistency of these effects across other cell lines (Figure S1C–G) collectively indicates that cycloastragenol acts as a potent suppressor of EMT activation.

### 3.6 Cycloastragenol Directly Targets PI3K and Inhibits the PI3K/AKT Pathway in Colorectal Cancer Cells

**FIGURE 7:**
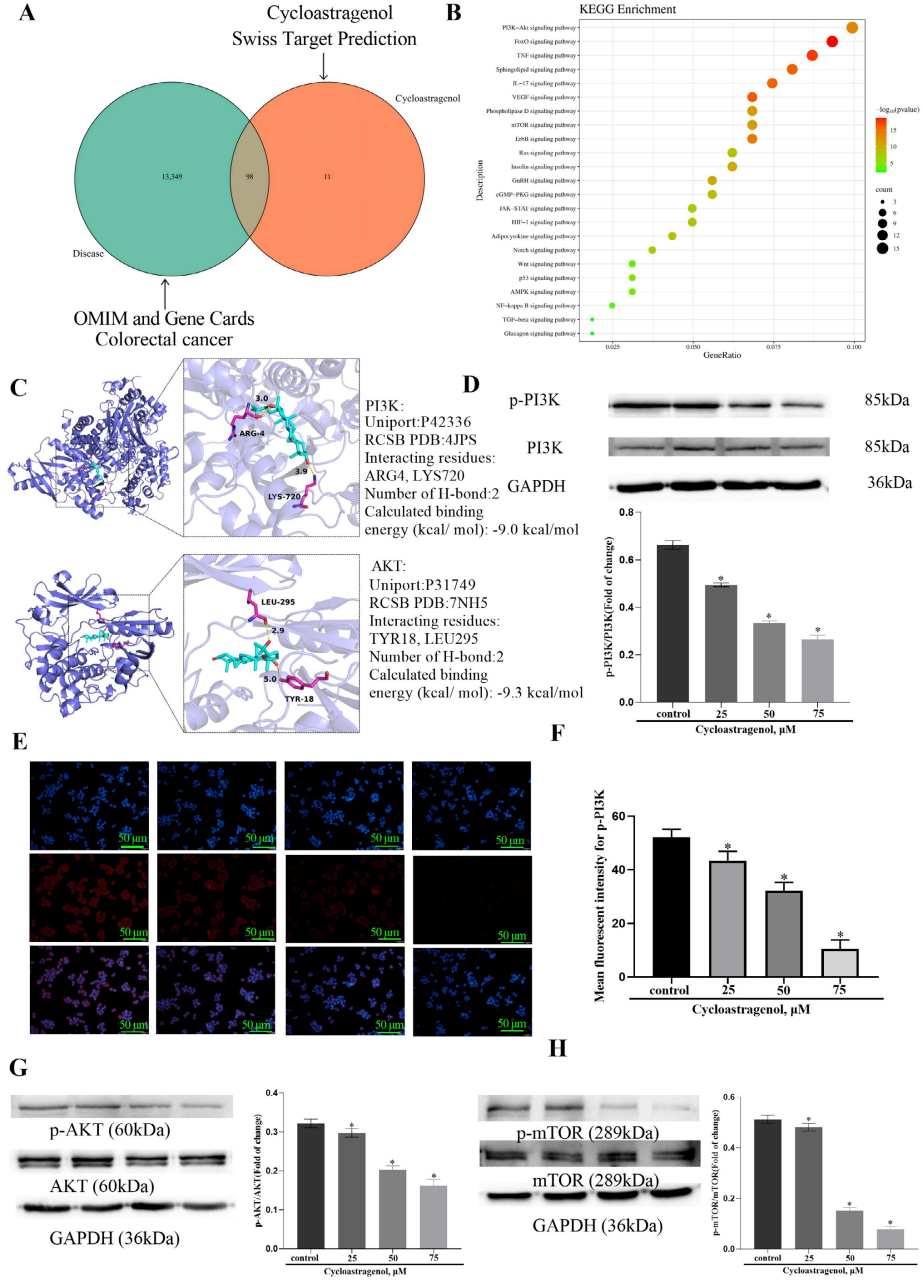
Cycloastragenol directly targets PI3K and inhibits PI3K/AKT pathway in colorectal cancer cells. (A) Veen diagram of common gene targets of colorectal cancer and cycloastragenol. (B) Signalling pathways enriched terms in KEGG pathways. (C) Molecular docking analysis of NC targeting PI3K or AKT. (D) Western blot was used to detect expression levels of p‐PI3K, PI3K. (E) and (F) Immunofluorescence analysis was used to detect expression levels of p‐PI3K. (G, H) Western blot was used to detect expression levels of p‐AKT, AKT, p‐mTOR, mTOR. Data were expressed as mean ± SD, *n* = 3. Data are presented as the mean ± SD. *p < 0.05 vs. the control group, Scale bars = 50 μm.

To further elucidate the mechanism by which cycloastragenol inhibits epithelial–mesenchymal transition (EMT), we performed a comprehensive network pharmacology analysis. As illustrated in Figure 7A, the intersection of predicted targets for colorectal cancer and Cycloastragenol identified 98 common targets. Subsequently, KEGG pathway enrichment analysis was performed using the DAVID database. This analysis identified 23 significant signalling pathways, including the PI3K/AKT signalling pathway (Figure 7B). The PI3K/AKT signalling axis serves as a central regulator in oncogenesis, critically driving tumour cell proliferation and invasion. Its pivotal role is further demonstrated by its capacity to initiate EMT [29]. To further investigate the binding affinity of cycloastragenol with proteins associated with the PI3K‐AKT signalling pathway, a molecular docking analysis was conducted using the Autodock software. The binding activities between cycloastragenol and two key proteins (PI3K and AKT) were analysed.

Molecular docking analysis predicted that cycloastragenol may interact with PI3K (interacting site: ARG4, LYS720; interactions: hydrogen bond interaction; binding energies: −9.0 kcal/mol) and AKT (interacting site: TYR18, LEU295; interactions: hydrogen bond interaction; binding energies: −9.3 kcal/mol) (Figure 7C), suggesting a potential binding mode that warrants further experimental validation. Through western blot analysis, we demonstrated that cycloastragenol significantly inhibited the expression levels of p‐PI3K, p‐AKT and p‐mTOR in a dose‐dependent manner (Figure 7D,G,H). Immunofluorescence experiments also confirmed that cycloastragenol inhibited the expression of PI3K (Figure 7E,F). Overall, these results indicate that cycloastragenol can effectively suppress the PI3K/Akt signalling pathway in colorectal cancer cells through direct interaction with PI3K.

### 3.7 The Metastasis Inhibited by Cycloastragenol Was Dependent on the Suppression of the PI3K/AKT Pathway

**FIGURE 8:**
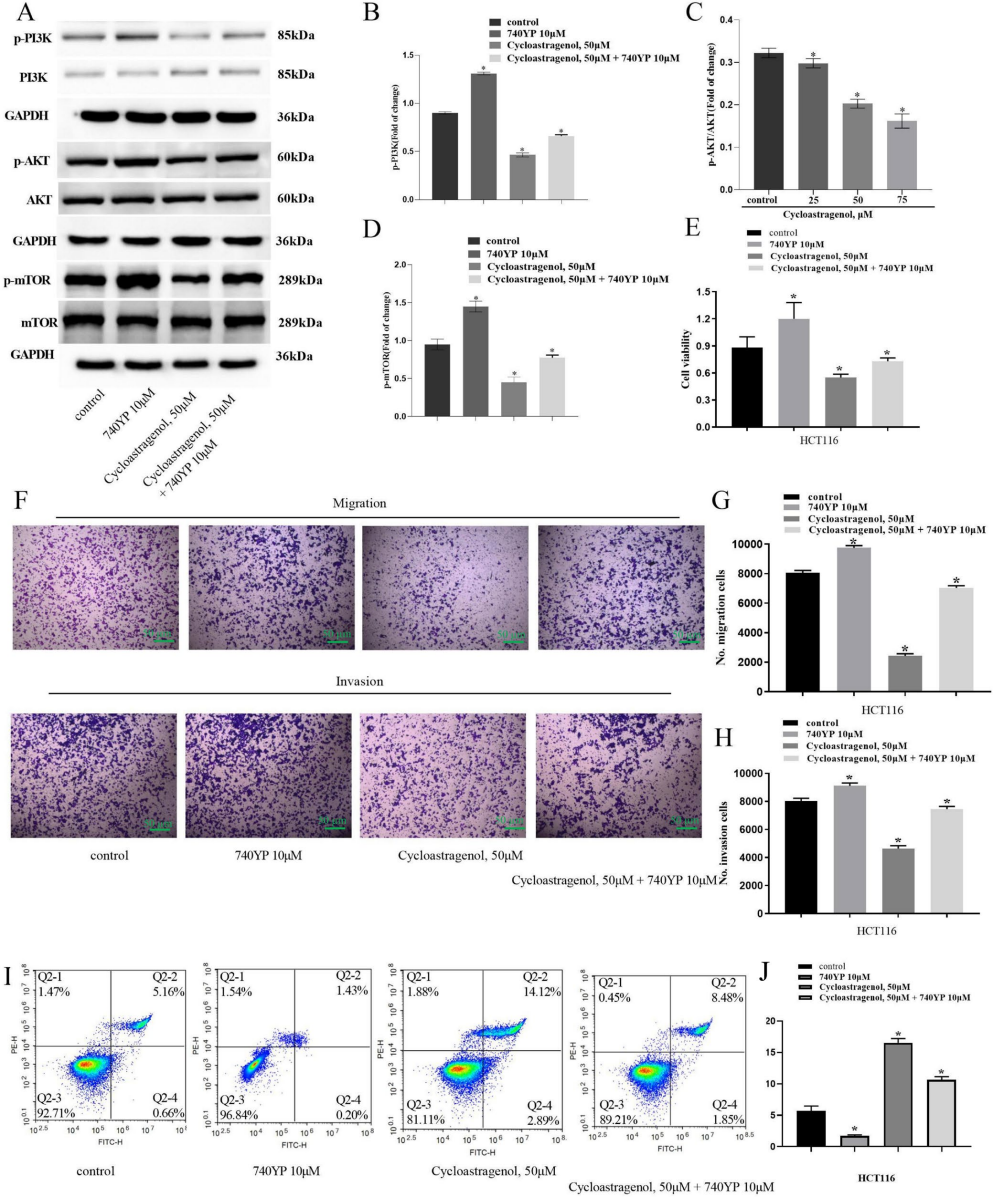
Anti‐tumour effects of cycloastragenol were dependent on the suppression of the PI3K/AKT pathway. The cells in the control group were treated with 0.1% DMSO for 24 h. (A–D) Western blot analysis showed that after treating the cells with 740 Y‐P, the levels of p‐PI3K/PI3K, p‐AKT/AKT, and p‐mTOR/mTOR were significantly increased. (E–J) 740 Y‐P could reverse the effect of cycloastragenol on the proliferation, migration, invasion and apoptosis of HCT116 cells. Data are presented as the mean ± SD. ^*^indicates a difference compared to the control group (*p* < 0.05), *n* = 3. Scale bars = 50 μm.

To mechanistically dissect the role of PI3K/Akt signalling in cycloastragenol‐mediated metastasis suppression, we employed both pharmacological and genetic approaches. The concentration of cycloastragenol (50 μM) was determined based on its IC_50_ value. The PI3K activator 740Y‐P significantly rescued cycloastragenol‐induced inhibition of PI3K, AKT and mTOR phosphorylation (Figure 8A–D). Consequently, 740Y‐P reversed the anti‐proliferative (Figure 8E), anti‐migratory (Figure 8F,G), anti‐invasive (Figure 8F,H) and pro‐apoptotic (Figure 8I,J) effects of cycloastragenol in colorectal cancer cells. Complementarily, siRNA‐mediated PI3K knockdown markedly abrogated the phosphorylation of PI3K, AKT and mTOR (Figure S2A–C). Furthermore, PI3K silencing synergistically enhanced cycloastragenol's suppression of proliferation (Figure S2D), migration (Figure S2E,F) and invasion (Figure S2G,H), as well as its promotion of apoptosis (Figure S2I,J). Collectively, these complementary experiments establish that cycloastragenol exerts its metastasis‐inhibitory effects primarily through inactivation of the PI3K/Akt pathway.

## 4 Discussion

Colorectal cancer (CRC) is one of the most prevalent malignant neoplasms globally. In recent years, the incidence of CRC has shown an annual increase and a trend towards younger patient populations. Approximately 20% of patients are diagnosed with metastatic disease at the time of initial diagnosis. The malignant proliferation and migration of CRC cells are associated with poor patient outcomes, and metastasis remains a critical factor contributing to the low survival rates in CRC. According to the National Cancer Institute of the United States, the 5‐year survival rate for patients with localised colorectal cancer is 90.6%, whereas it is only 14.7% for those with metastatic disease [30]. Cycloastragenol has been reported to exert anti‐tumour effects across multiple cancer types through distinct signalling cascades. In non‐small cell lung cancer, cycloastragenol induces apoptosis and protective autophagy via activation of the AMPK/ULK1/mTOR axis [14], whereas in gastric cancer, it negates constitutive STAT3 phosphorylation and potentiates paclitaxel‐induced apoptosis through suppression of JAK1/2 and Src kinases [15]. These findings suggest that the mechanistic action of cycloastragenol is highly context‐dependent, likely influenced by tumour type, mutational landscape and baseline pathway activity. In the present study, we demonstrate for the first time that cycloastragenol suppresses epithelial–mesenchymal transition and metastasis in colorectal cancer via inhibition of the PI3K/AKT signalling pathway. This distinct mechanism not only expands the current understanding of cycloastragenol's pleiotropic anti‐tumour properties but also underscores the importance of tumour‐specific therapeutic strategies when evaluating natural product‐based agents.

Epithelial–mesenchymal transition (EMT) is a critical biological process in cancer metastasis, facilitating the conversion of epithelial cells from a polarised, adherent phenotype to a motile, mesenchymal‐like state. This transition involves the loss of cell–cell adhesion molecules (e.g., E‐cadherin) and the acquisition of mesenchymal markers (e.g., N‐cadherin, vimentin), thereby enhancing cellular migratory and invasive properties [31, 32]. Under pathological conditions such as cancer, EMT is aberrantly activated, typically characterised by down‐regulation of E‐cadherin and upregulation of N‐cadherin and other mesenchymal markers (e.g., vimentin, Snail, Slug). In this study, we observed that cycloastragenol effectively modulated EMT‐related protein expression: it upregulated E‐cadherin while down‐regulating N‐cadherin, vimentin, Snail and Slug. These findings collectively demonstrate that cycloastragenol suppresses EMT activation, suggesting its potential as a therapeutic agent for inhibiting cancer metastasis.

The PI3K/AKT/mTOR signalling pathway plays a critical role in various biological processes and is often dysregulated in tumours, rendering it an important therapeutic target for treatment [33]. The potential involvement of the PI3K‐AKT signalling pathway in mediating cycloastragenol's effects on EMT has not yet been elucidated. Through network pharmacology analysis, we identified the enrichment of the PI3K/Akt signalling pathway. EMT can be activated by various signalling molecules and pathways upstream, such as the PI3K/Akt pathways [34].

We first indicated the molecular space binding potential of cycloastragenol with PI3K (binding energies: −9.0 kcal/mol) and AKT (binding energies: −9.3 kcal/mol). Based on these findings, we investigated the PI3K/Akt pathway to elucidate the underlying mechanism through which cycloastragenol inhibits the EMT process in colorectal cancer cells. We found that cycloastragenol could inhibit the phosphorylation of PI3K, AKT and mTOR in a dose‐dependent manner. Furthermore, upon administration of the PI3K activator 740YP, we observed that it could effectively reverse the anti‐tumour effects of cycloastragenol on colorectal cancer cells. These findings demonstrate that the metastasis‐inhibitory effects of cycloastragenol in colorectal cancer cells are mediated through the PI3K/Akt signalling pathway.

Several limitations of this study should be noted. First, experimental manipulations were limited to PI3K knockdown and overexpression, without corresponding gain‐ or loss‐of‐function validation of AKT or mTOR. Although the current findings provide preliminary evidence supporting the involvement of the PI3K/AKT pathway in cycloastragenol‐mediated suppression of colorectal cancer metastasis, the potential contribution of other signalling cascades (e.g., AMPK, STAT3) cannot be excluded. Second, further investigations employing more rigorous experimental designs are warranted to delineate the precise mechanisms and specific roles of these molecules in the observed anti‐metastatic effects.

## 5 Conclusion

**FIGURE 9:**
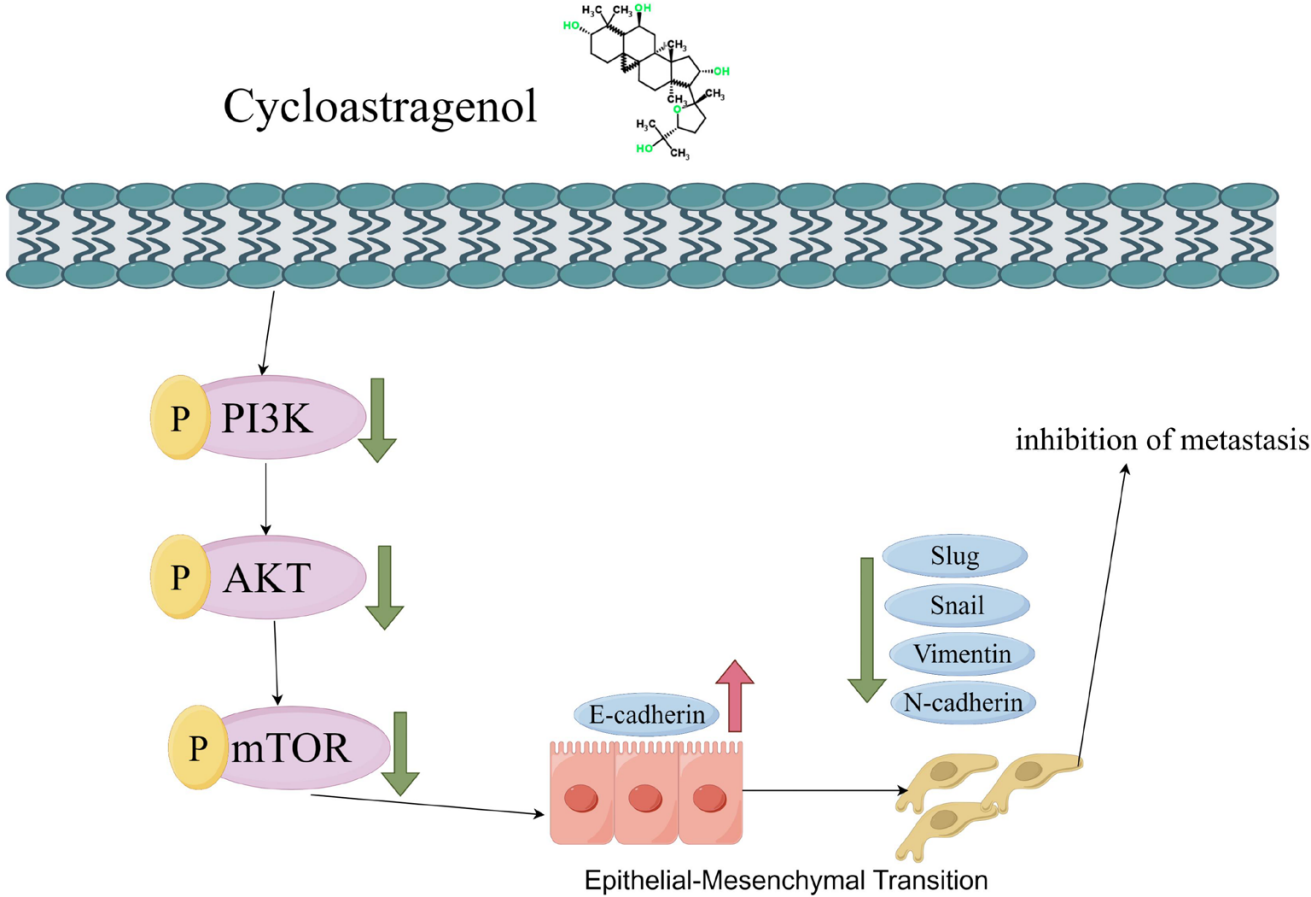
Schematic of cycloastragenol modulates metastasis and EMT in colorectal cancer cells via PI3K/AKT/mTOR pathways.

This study provides converging evidence from both in vitro and in vivo settings that cycloastragenol curbs colorectal tumour growth and metastasis. The anti‐tumour efficacy is mechanistically linked to its capacity to block EMT progression by targeting the PI3K‐AKT signalling axis (Figure 9). These findings establish a preclinical rationale for developing cycloastragenol as a promising candidate drug for CRC, with potential implications for mitigating disease burden and improving therapeutic outcomes.

## Author Contributions

**PanFeng Feng:** conceptualization, methodology, data curation, investigation. **JingRong Miao:** conceptualization, methodology, data curation, investigation.

## Funding

This work was supported by Jiangsu Pharmaceutical Association—Yaoyanxinsheng Fund, 202564030, Jiangsu Pharmaceutical Association—Tianqing Fund, T202512, Scientific Research Project of Nantong Municipal Health and Family Planning Commission, MS2024038, Jiangsu Key Laboratory of New Drug Research and Clinical Pharmacy fund, 25KF03, Nantong Pharmaceutical Association Fund, (ntyxky2509, ntyxky2524), Guangzhou Zhiyi Charity Foundation fund, NA, Nantong Science and Technology Program Project, MSZ2025093, and Fund of Drug Policy and Pharmaceutical Care of Nantong City, 2023NTPA07.

## Conflicts of Interest

The authors declare no conflicts of interest.

## Supporting information


**Data S1:** jcmm71128‐sup‐0001‐DataS1.pdf.

**Figure S1‐S2:** jcmm71128‐sup‐0002‐FigureS1‐S2.doc.

**Figure S1:** jcmm71128‐sup‐0003‐FigureS1.pdf.

## Data Availability

The original data that support the findings of this study are available from the corresponding author, upon reasonable request.
