# Supplementary material for: Cycloastragenol Inhibits Colorectal Cancer Cell Metastasis via Epithelial–Mesenchymal Transition and the PI3K Signalling Pathway
Source: J Cell Mol Med. 2026 Apr 16;30(8):e71128. doi: 10.1111/jcmm.71128 (PMC13086017; doi:10.1111/jcmm.71128)
Supplement: Supplementary file 1 — Data S1: jcmm71128‐sup‐0001‐DataS1.pdf. [file JCMM-30-e71128-s002.pdf]

CBTCCCAS Certificate of STR Analysis

1.Sample

DLD-1

2.Method and Procedure

Sample DNA was extracted by Microread Genomic DNA Kit.
PCR was amplified with STR Multi-amplification Kit(MicroreaderTM21 ID System).
PCR products were assayed with ABI 3730xl DNA Analyzer(Applied Biosystems®).
Data were analyzed using GeneMapperID-X software and then compared with the ATCC and DSMZdatabases for reference matching.

3.Results

D5S818	13
D13S317	8, 11
D7S820	10, 12
D16S539	12, 13
vWA	18, 19
TH01	7, 9. 3
Amelogenin	X, Y
TPOX	8, 11
CSF1PO	11, 12

The above results were consistent with the DNA profiles reported by ATCC, DSMZ and JCRB, and indicated no other human cell lines contamination.

Cell Bank,
Type Culture Collection,
Chinese Academy of Sciences
(CBTCCCAS)

中国科学院典型培养物保藏委员会
细 胞 库

CBTCCCAS Certificate of STR Analysis

1.Sample

HCT 116

2.Method and Procedure

Sample DNA was extracted by Microread Genomic DNA Kit.
PCR was amplified with STR Multi-amplification Kit(MicroreaderTM21 ID System).
PCR products were assayed with ABI 3730xl DNA Analyzer(Applied Biosystems®).
Data were analyzed using GeneMapperID-X software and then compared with the ATCC and DSMZdatabases for reference matching.

3.Results

D5S818	10, 11
D13S317	10, 12
D7S820	11, 12
D16S539	11, 13
vWA	17, 22
TH01	8, 9
Amelogenin	X, Y
TPOX	8, 9
CSF1PO	7, 10

The above results were consistent with the DNA profiles reported by ATCC, and DSMZ, and indicated no other human cell lines contamination.

Cell Bank,
Type Culture Collection,
Chinese Academy of Sciences
(CBTCCCAS)

中国科学院典型培养物保藏委员会
细 胞 库

CBTCCCAS

Certificate of STR Analysis

1.Sample

SW620

2.Method and Procedure

Sample DNA was extracted by Microread Genomic DNA Kit.
PCR was amplified with STR Multi-amplification Kit(MicroreaderTM21 ID System).
PCR products were assayed with ABI 3730xl DNA Analyzer(Applied Biosystems®).
Data were analyzed using GeneMapperID-X software and then compared with the ATCC and DSMZdatabases for reference matching.

3.Results

D5S818	13
D13S317	12
D7S820	8, 9
D16S539	9, 13
vWA	16
TH01	8
Amelogenin	X
TPOX	11
CSF1PO	13, 14

The above results were consistent with the DNA profiles reported by ATCC, and DSMZ, and indicated no other human cell lines contamination.

Cell Bank,
Type Culture Collection,
Chinese Academy of Sciences
(CBTCCCAS)

中国科学院典型培养物保藏委员会
细胞库
