# Supplementary material for: Cycloastragenol Inhibits Colorectal Cancer Cell Metastasis via Epithelial–Mesenchymal Transition and the PI3K Signalling Pathway
Source: J Cell Mol Med. 2026 Apr 16;30(8):e71128. doi: 10.1111/jcmm.71128 (PMC13086017; doi:10.1111/jcmm.71128)
Supplement: Supplementary file 2 — Figure S1‐S2: jcmm71128‐sup‐0002‐FigureS1‐S2.doc. [file JCMM-30-e71128-s001.doc]

Supplemental Figure 1


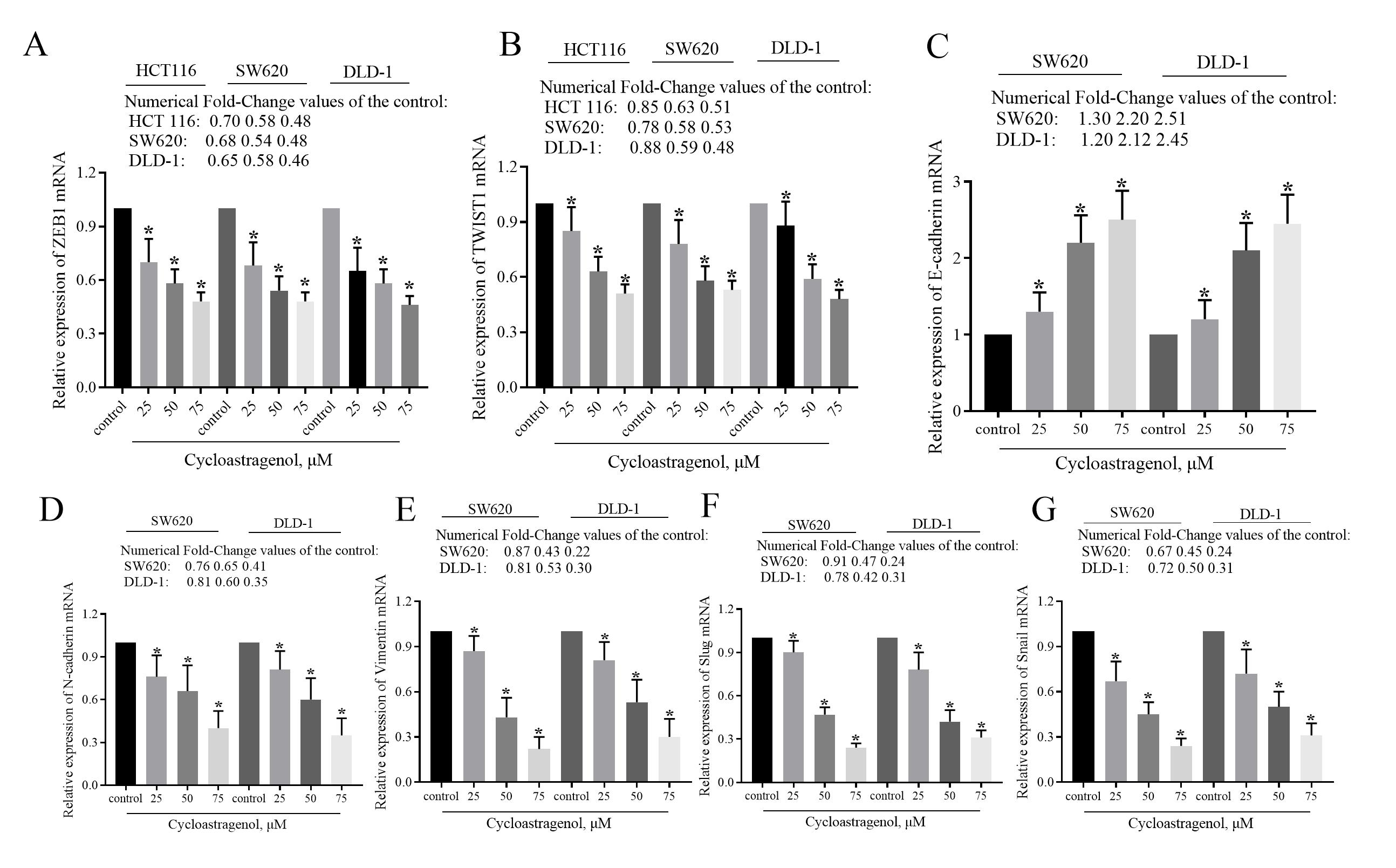


Fig.1 Effects of Cycloastragenol on the mRNA expression of EMT-related markers. A-B Relative mRNA expression of ZEBI and TWIST1 in HCT116, SW620 and DLD-1 cells. C-G Relative mRNA expression of E-cadherin, N-cadherin, Vimentin, slug and Snail in SW620 and DLD-1 cells. Data were expressed as mean ± SD, n = 3. Data are presented as the mean ± SD. *P < 0.05 vs. the control group.

Supplemental Figure 2


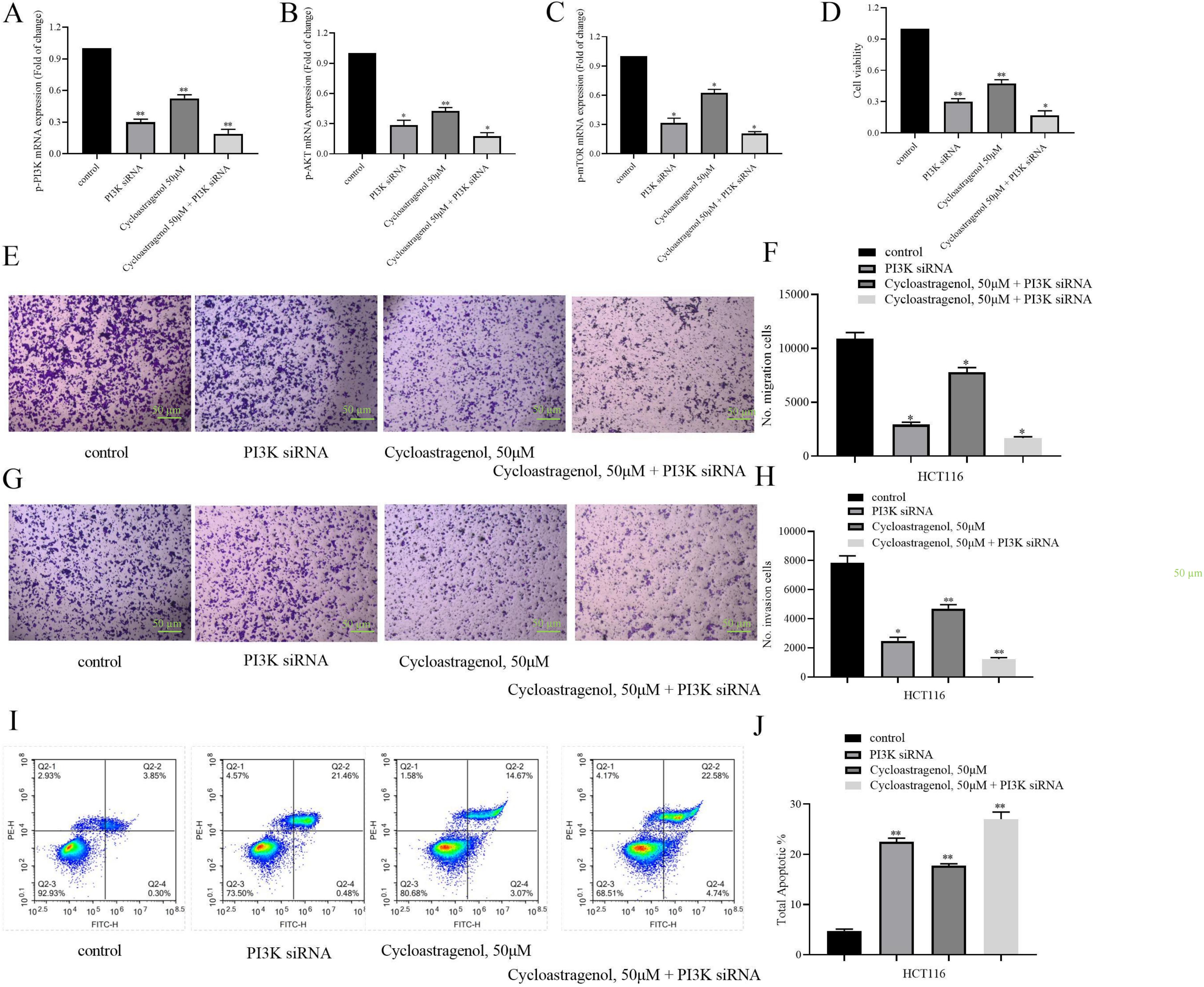


Fig.2. Knockdown of PI3K further confirmed that Cycloastragenol exerts its anti-tumor effects by inhibiting the PI3K-AKT-mTOR signaling pathway. The cells in control group and PI3K siRNA group were treated with 0.1% DMSO for 24 h. A-C RT-PCR analysis showed that after treating the cells with PI3K siRNA, the levels of p-PI3K/PI3K, p-AKT/AKT, and p-mTOR/mTOR were significantly decreased. D: The CCK-8 assay was employed to evaluate the impact of PI3K siRNA on cell viability. E-H: Transwell migration and invasion assay were used to detect the impact of PI3K siRNA on cell migration and invasion. I-J: Flow cytometric analysis of apoptosis was performed to assess the regulatory role of PI3K siRNA in cell apoptosis. Data are presented as the mean ± SD. *P < 0.05, **P < 0.01 vs. the control group.
