# Supplementary material for: Cycloastragenol Inhibits Colorectal Cancer Cell Metastasis via Epithelial–Mesenchymal Transition and the PI3K Signalling Pathway
Source: J Cell Mol Med. 2026 Apr 16;30(8):e71128. doi: 10.1111/jcmm.71128 (PMC13086017; doi:10.1111/jcmm.71128)

Fig1E: Ki67 358kDa

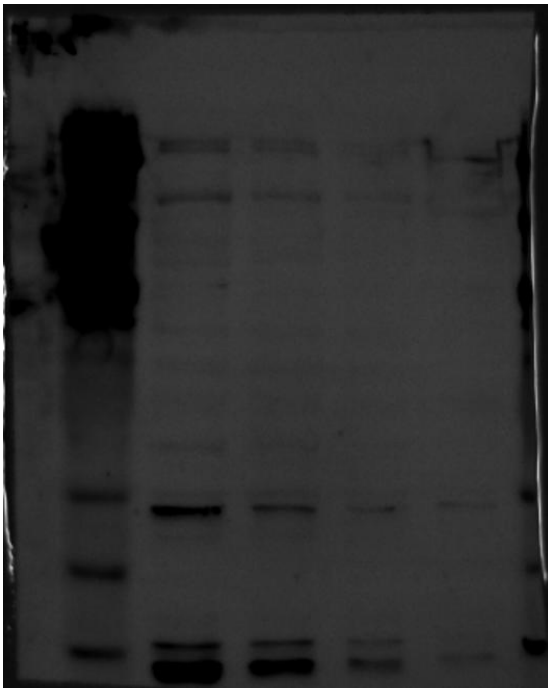

Fig1E: Ki67-GAPDH 36kDa

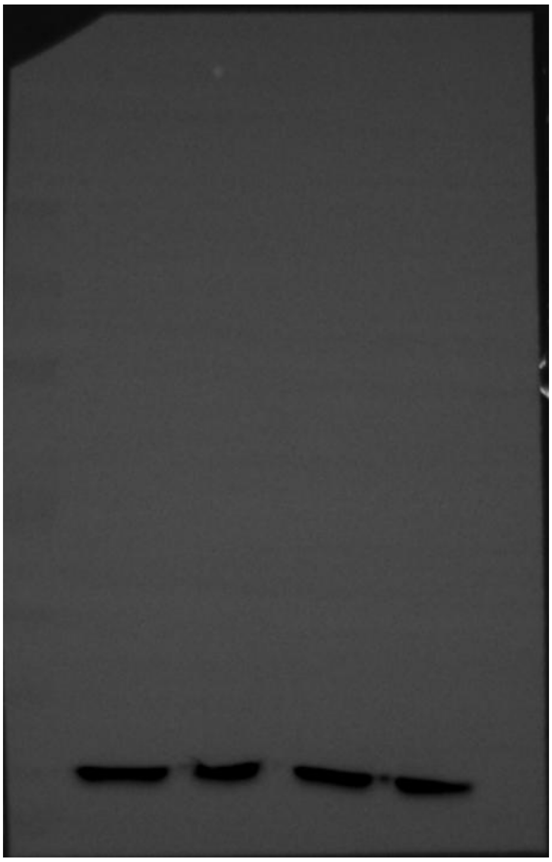

Fig1F: PCNA 29kDa

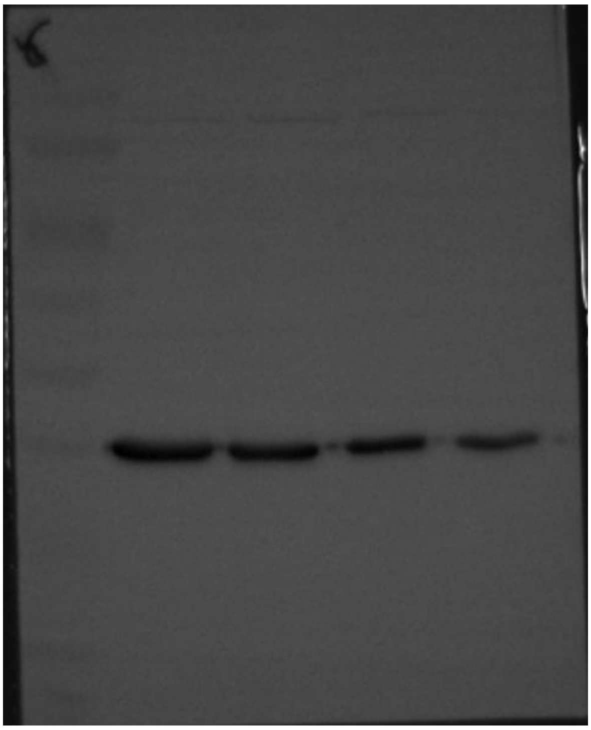

Fig1F: PCNA-GAPDH 36kDa

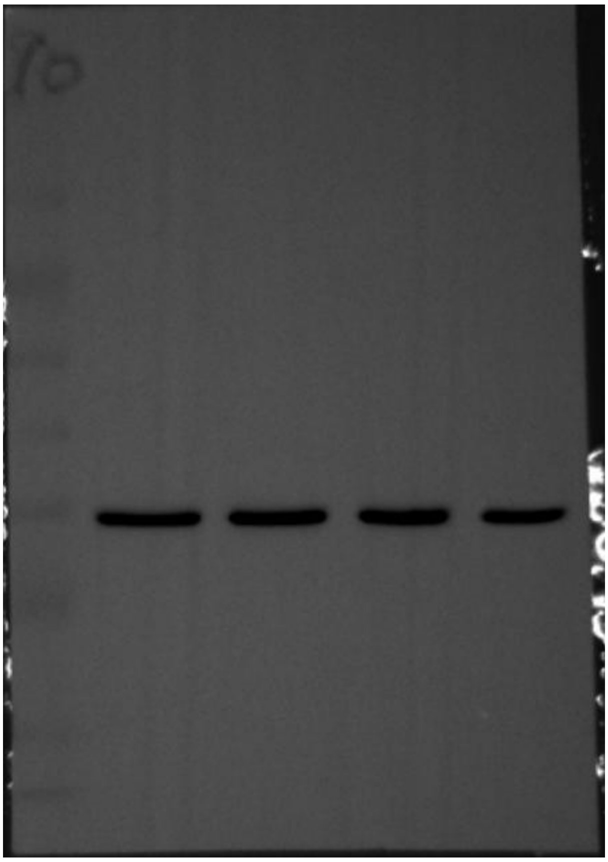

Fig4D: Bax 21kDa

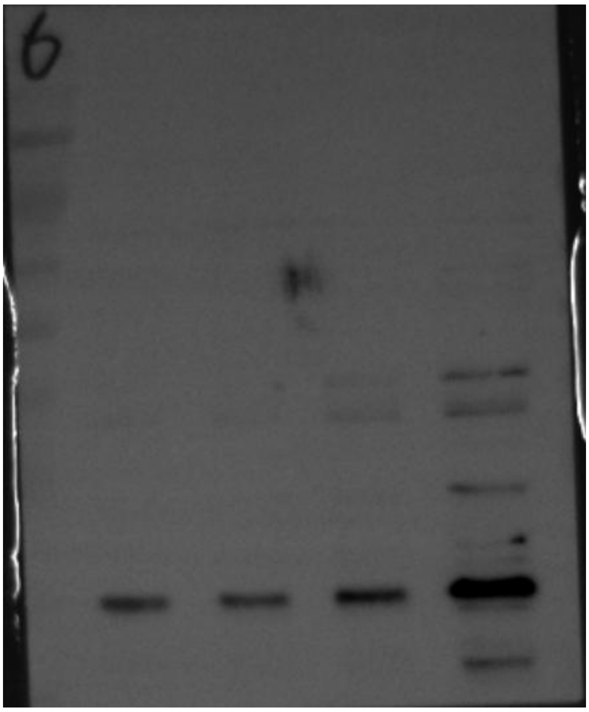

Fig4D: Bax-GAPDH 36kDa

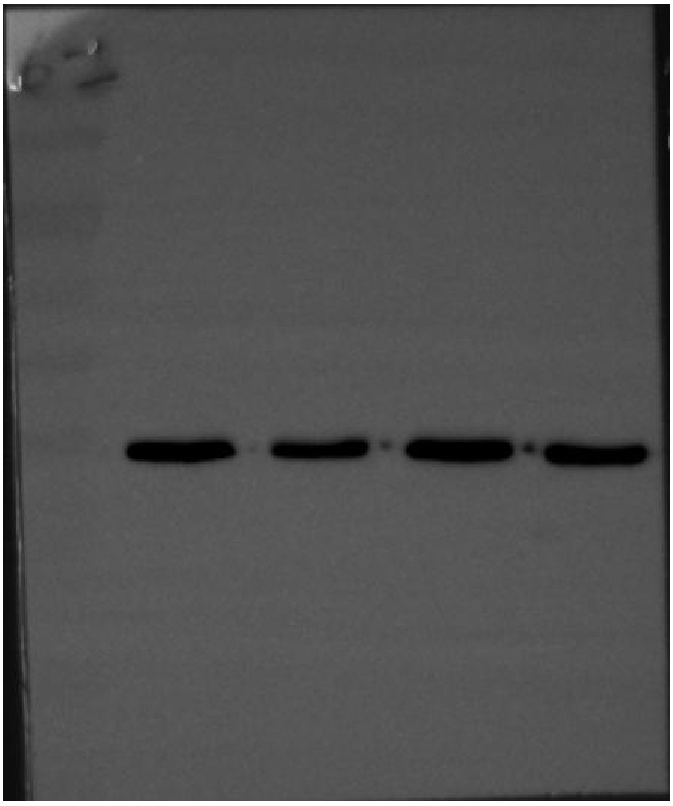

Fig4D: Bcl2 26kDa

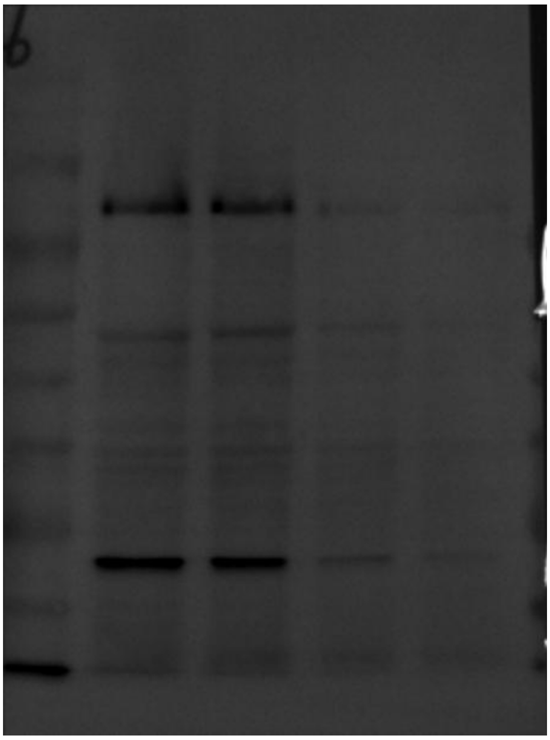

Fig4D: Bcl2-GAPDH 36kDa

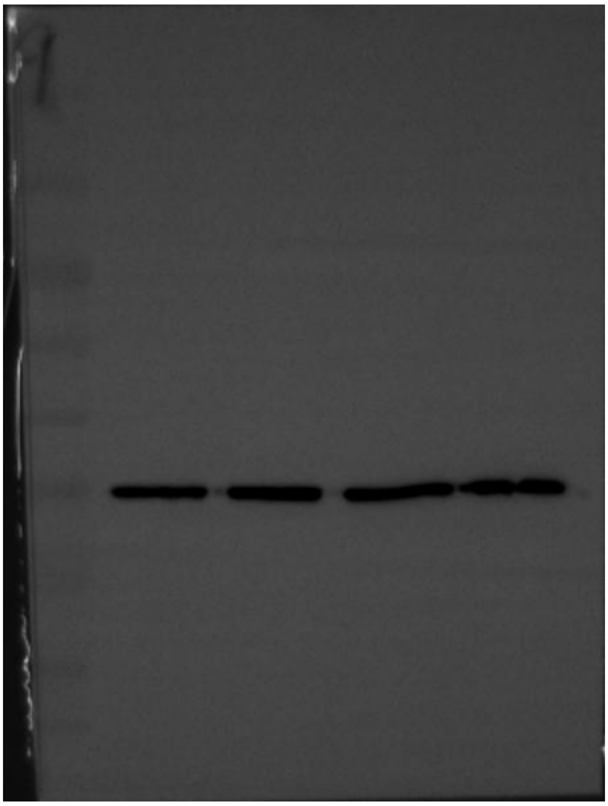

Fig4D: Cleaved caspase 3 19kDa

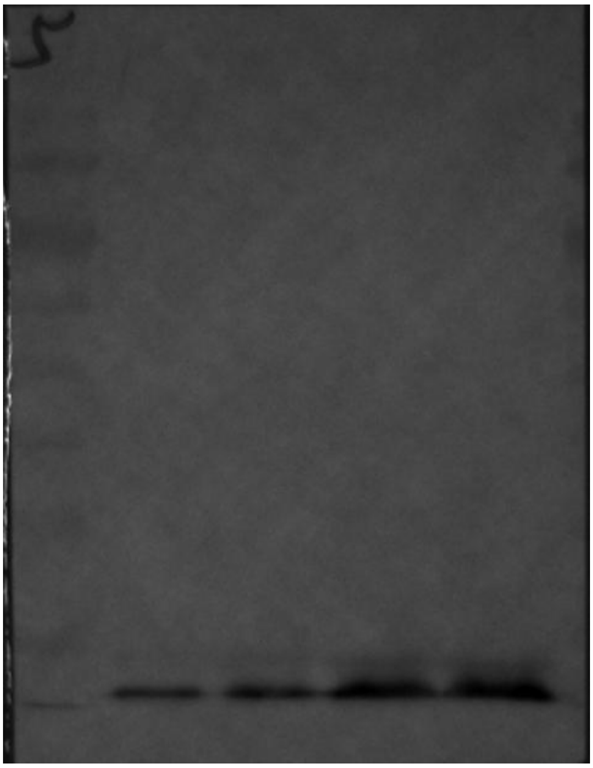

Fig4D: Cleaved caspase 3-GAPDH 36kDa

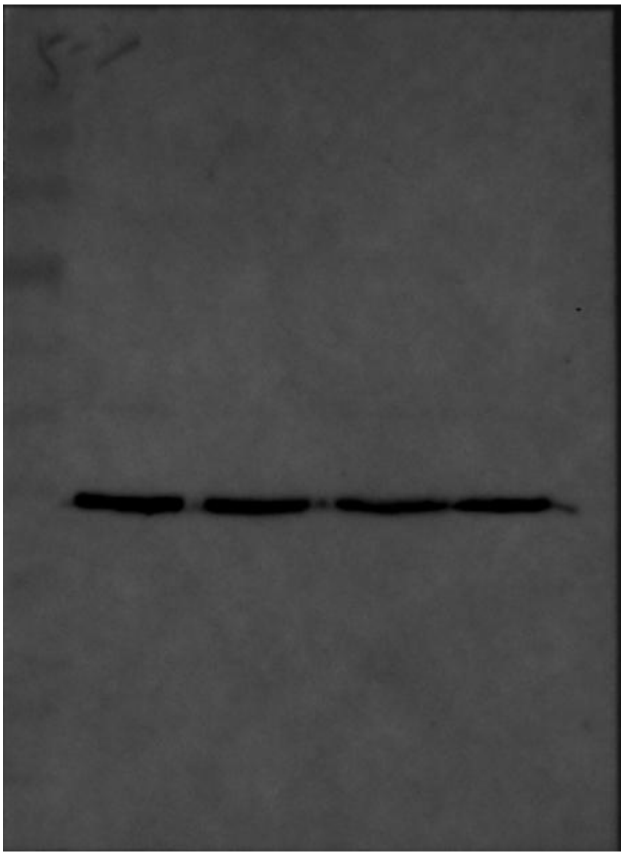

Fig6A: E-cadherin 130kDa

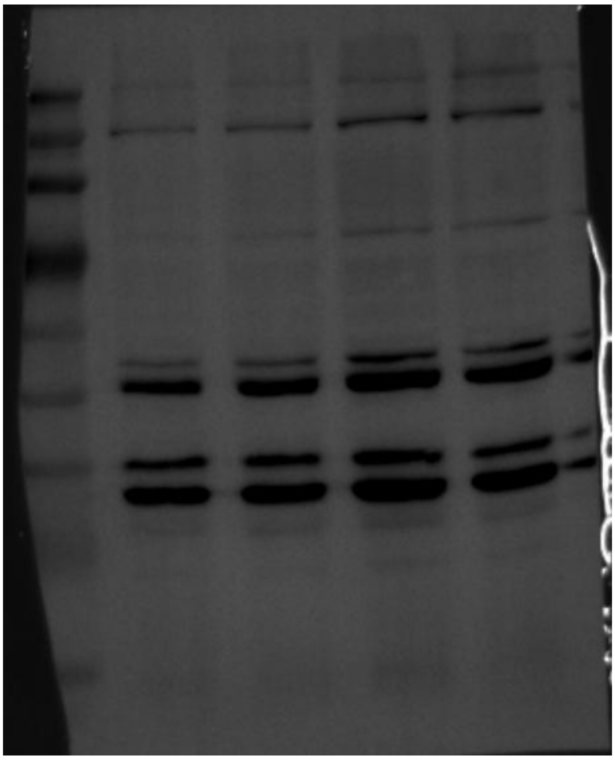

Fig6A: E-cadherin-GAPDH 36kDa

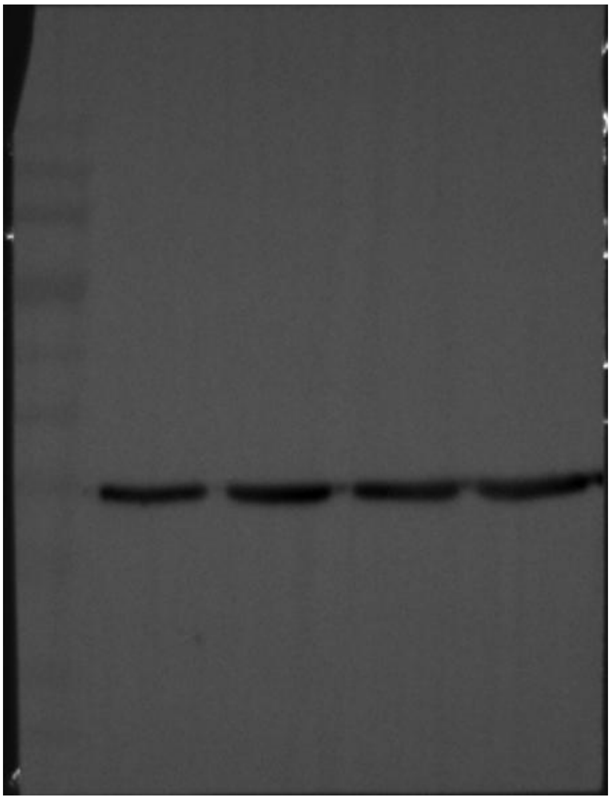

Fig6B: N-cadherin 125kDa

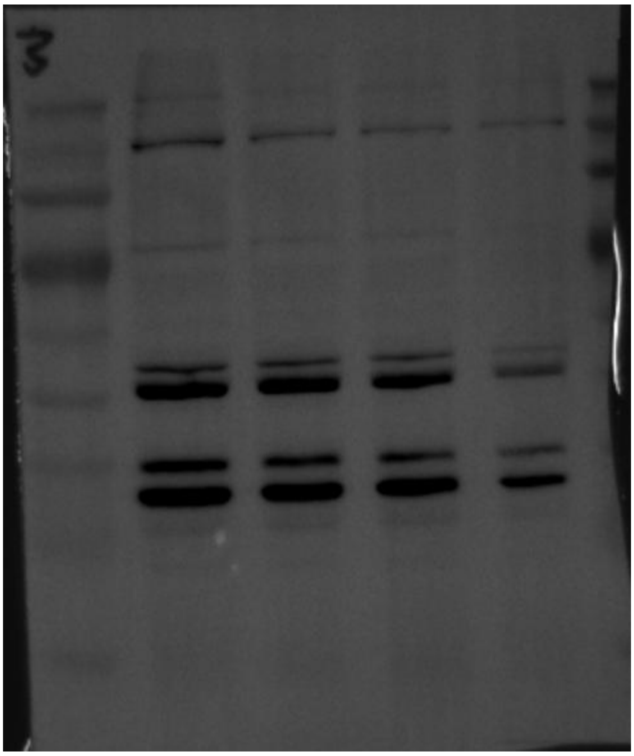

Fig6B: N-cadherin-GAPDH 36kDa

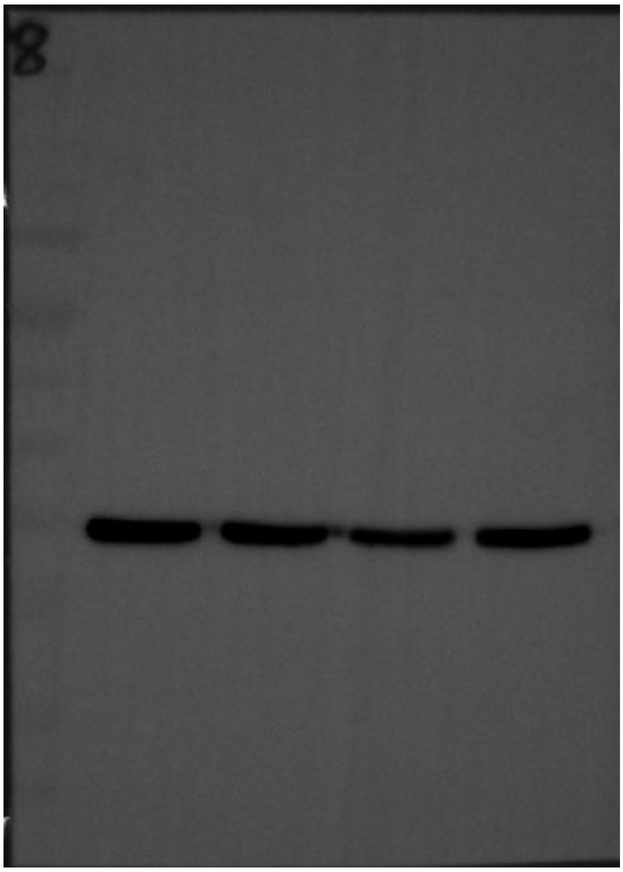

Fig6G:Vimentin 57kDa

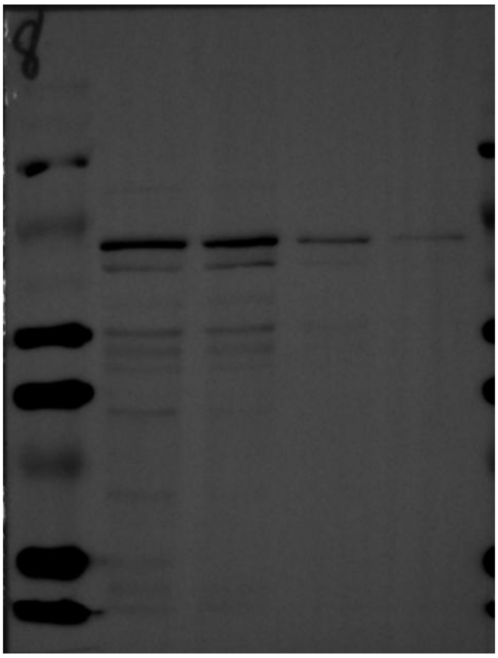

Fig6G:Vimentin-GAPDH 36kDa

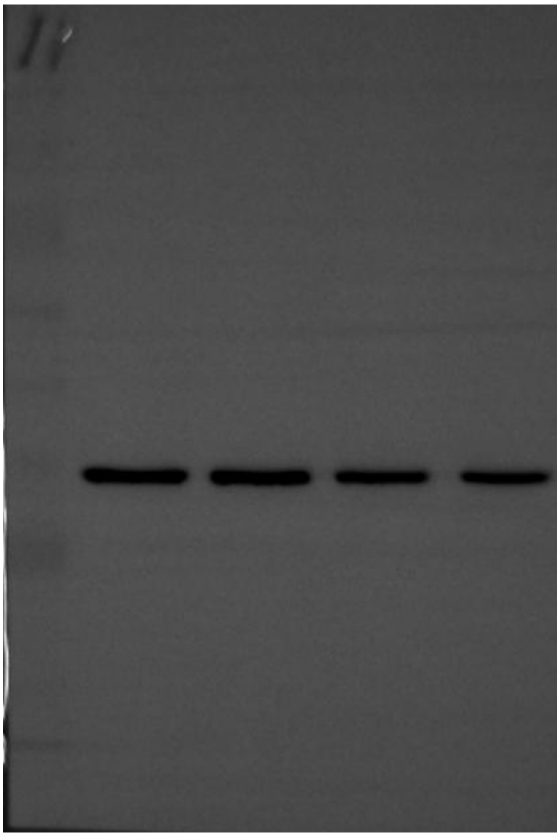

Fig6H:Slug 30kDa

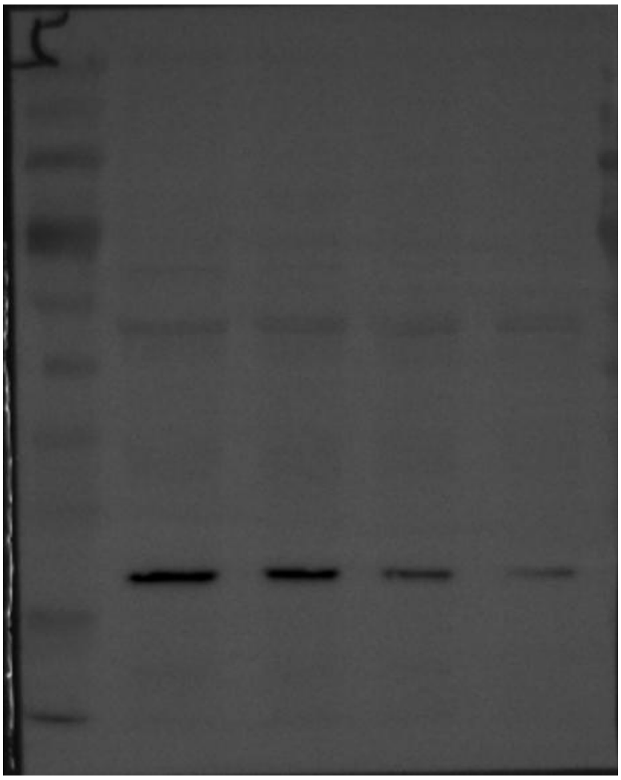

Fig6H:Slug-GAPDH 36kDa

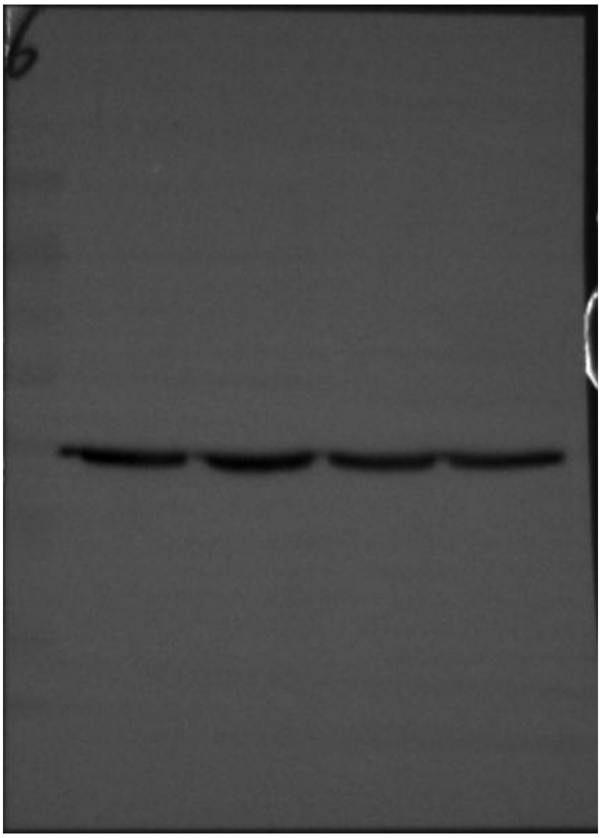

Fig6I:Snail 29kDa

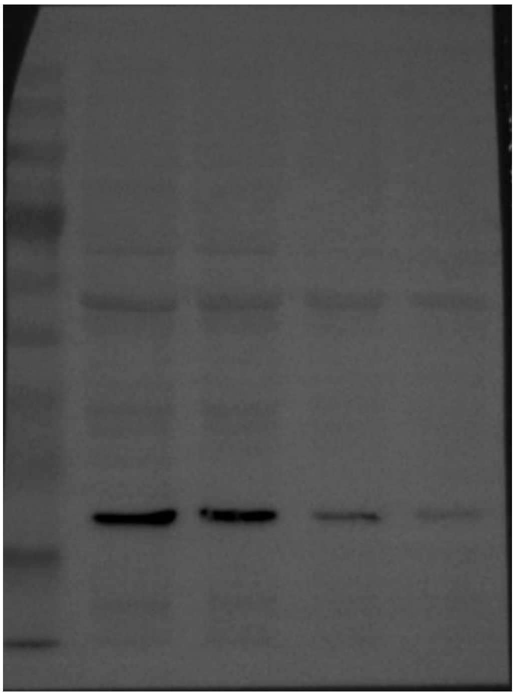

Fig6I:Snail-GAPDH 36kDa

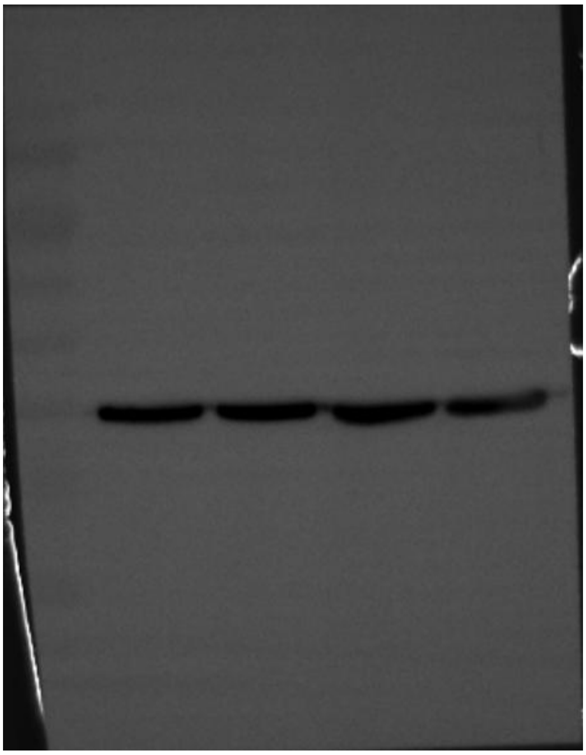

Fig7D:p-PI3K 85kDa

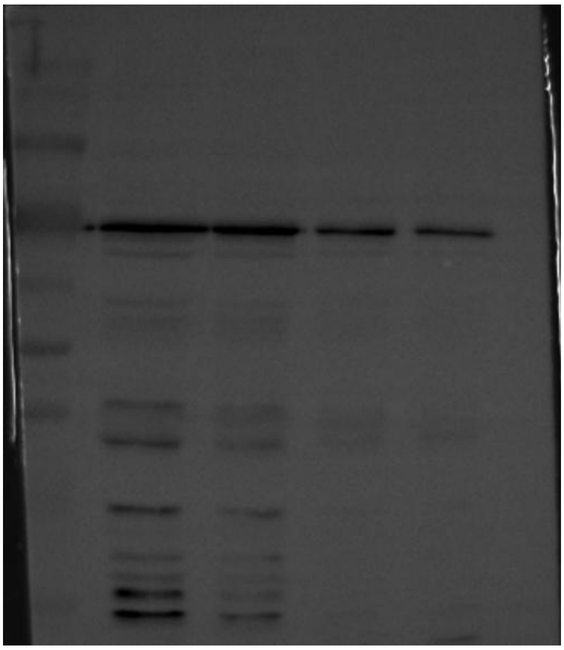

Fig7D:PI3K 85kDa

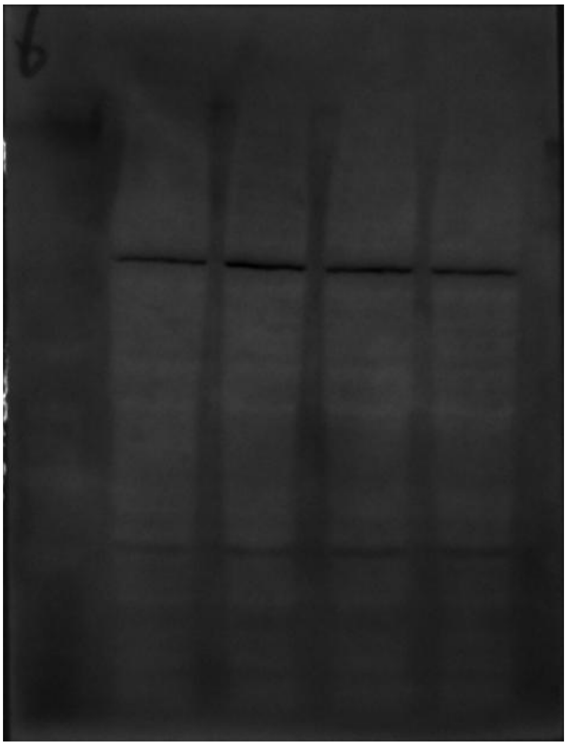

Fig7D:GAPDH 36kDa

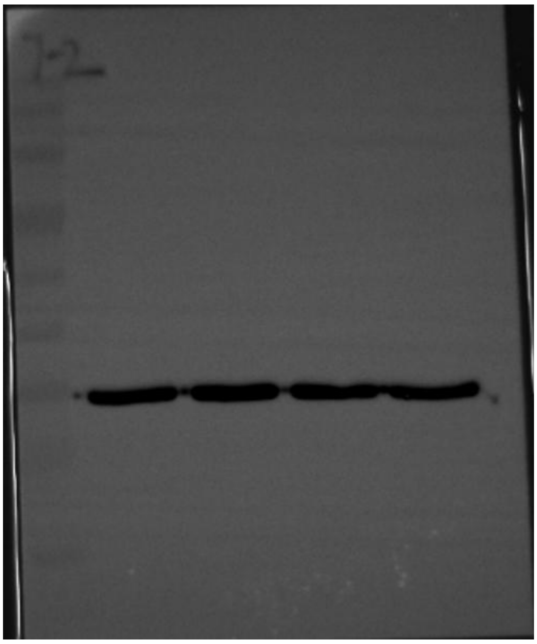

Fig7G:p-AKT 60kDa

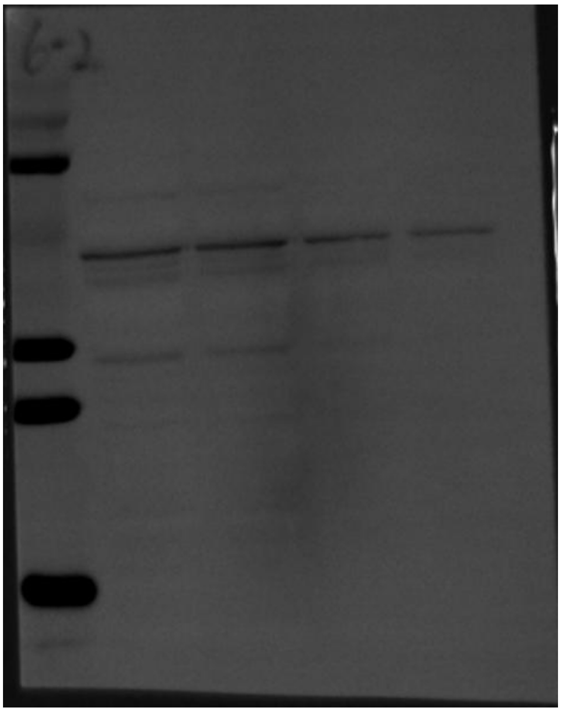

Fig7G:AKT 60kDa

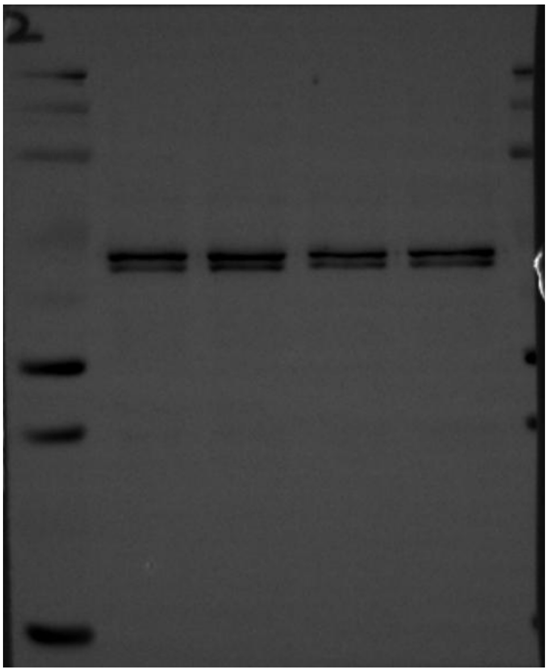

Fig7G:GAPDH 36kDa

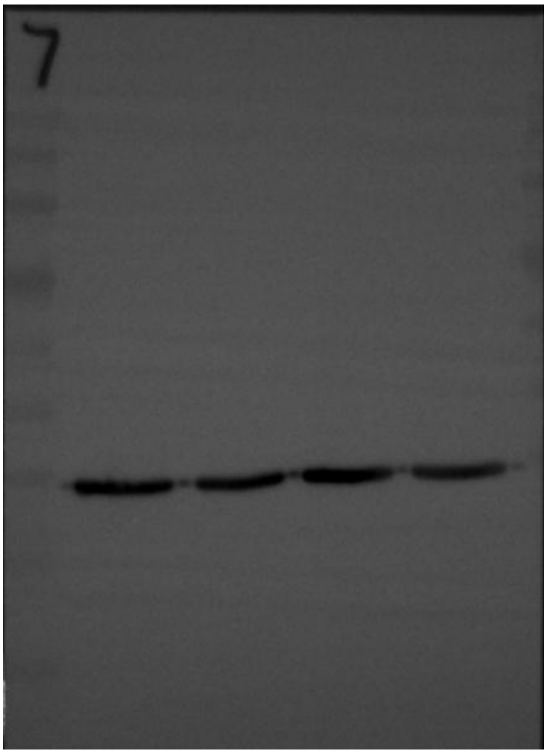

Fig7H:p-mTOR 289kDa

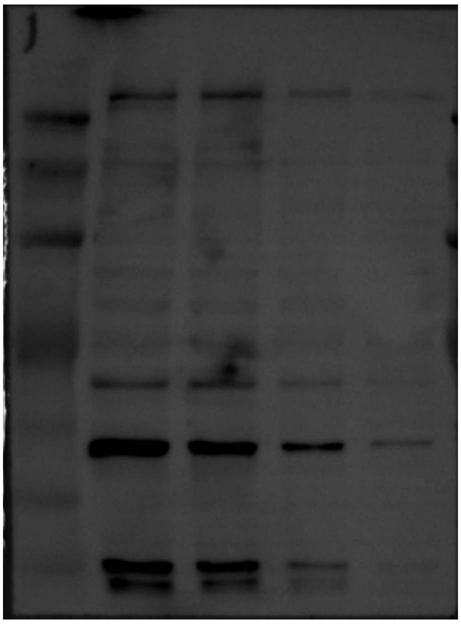

Fig7H:mTOR 289kDa

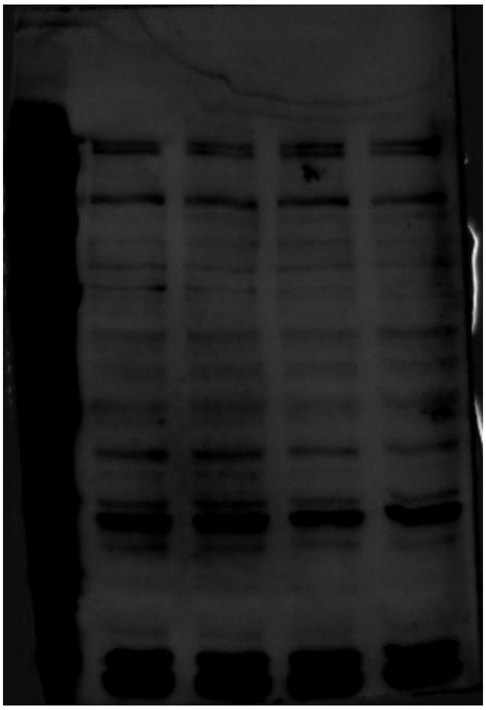

Fig7H:GAPDH 36kDa

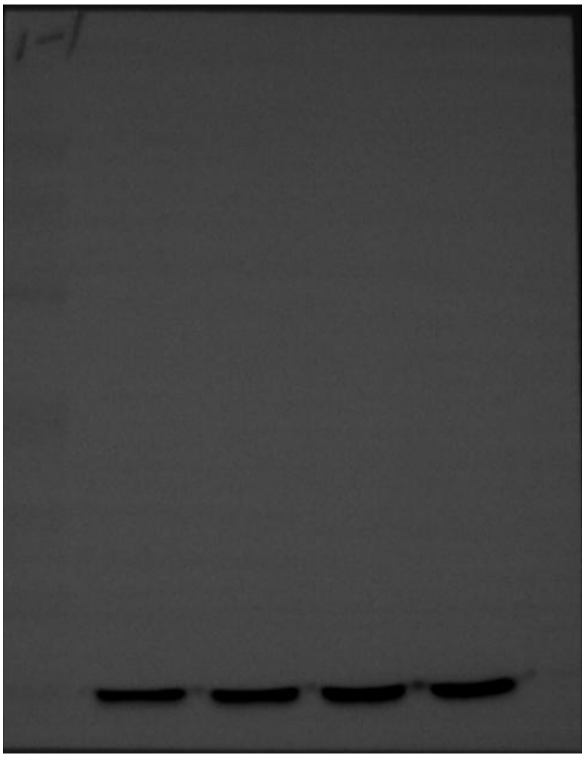

Fig8A:p-PI3K 85kDa

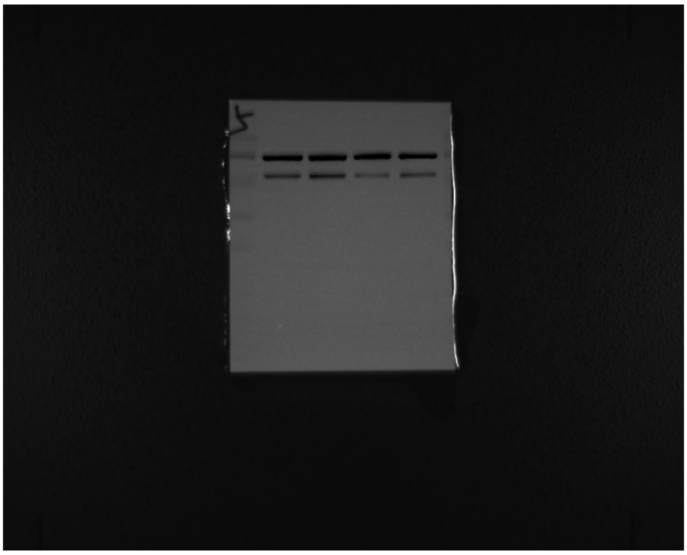

Fig8A:PI3K 85kDa

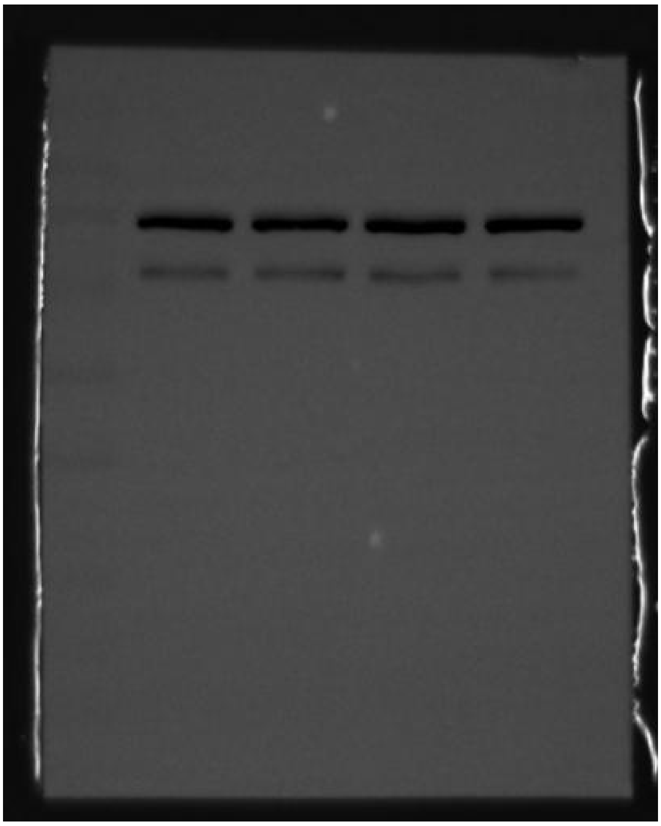

Fig8A:PI3K-GAPDH 36kDa

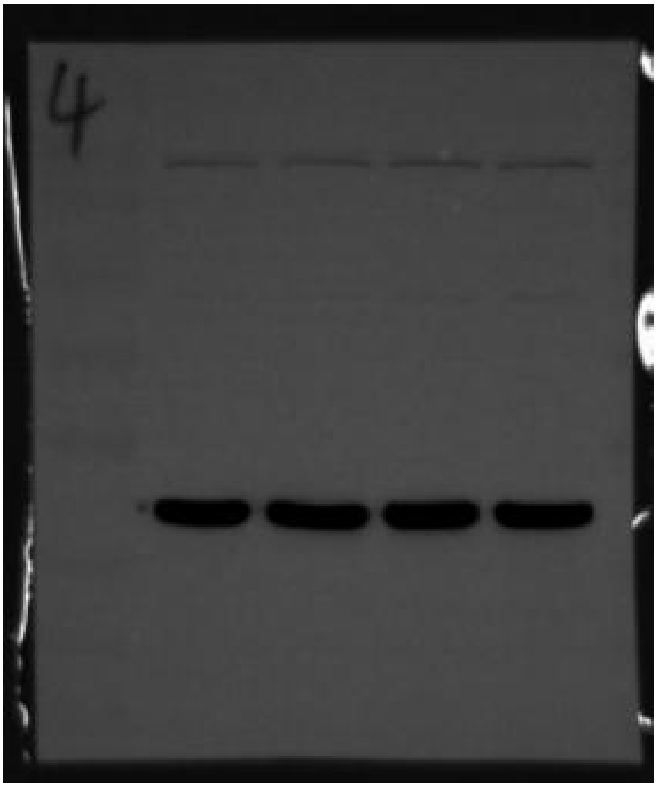

Fig8A:p-AKT 60kDa

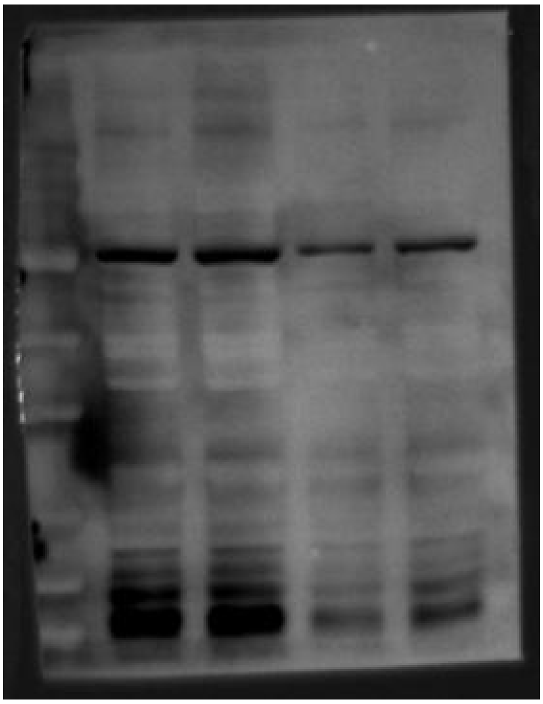

Fig8A:AKT 60kDa

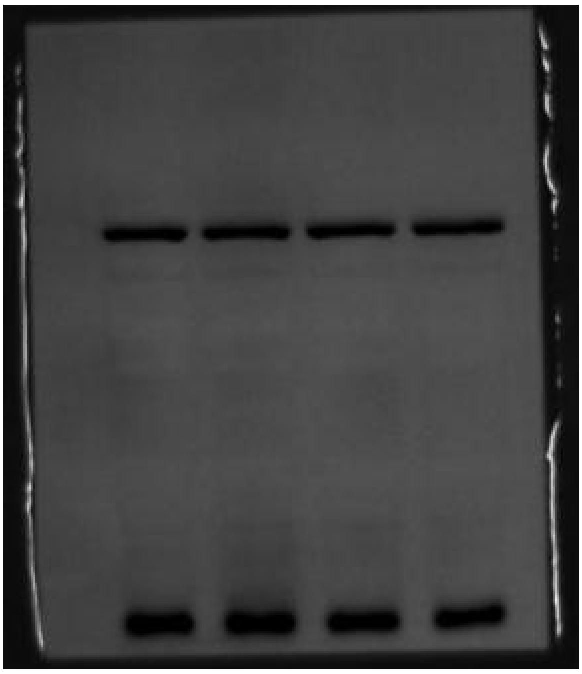

Fig8A:AKT-GAPDH 36kDa

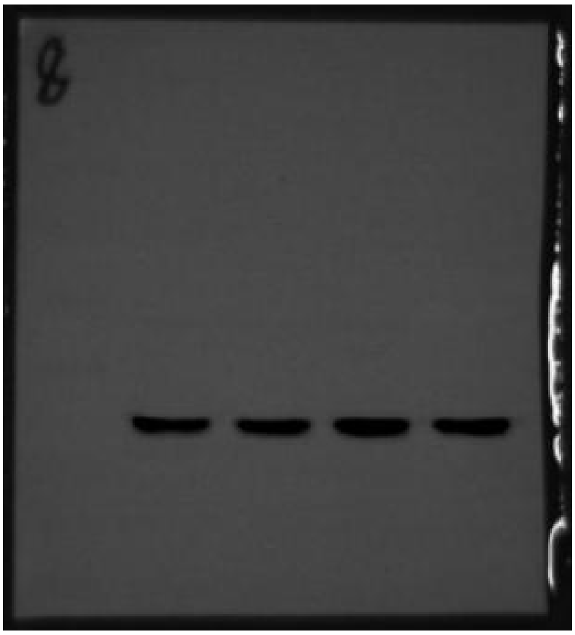

Fig8A:p-mTOR 289kDa

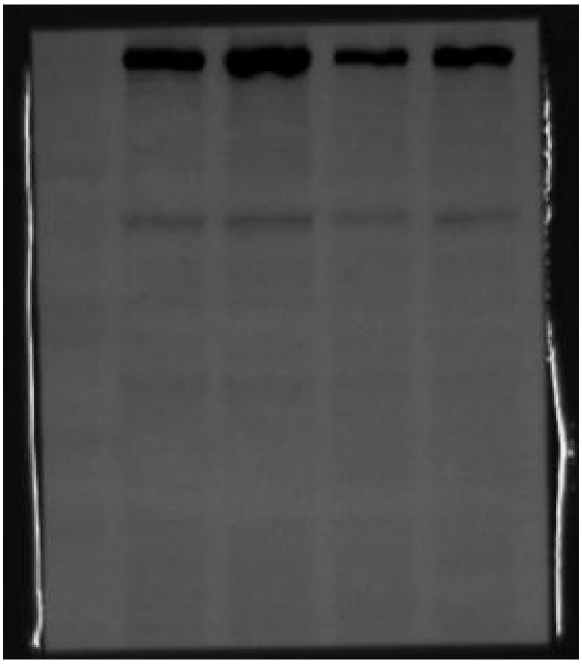

Fig8A:mTOR 289kDa

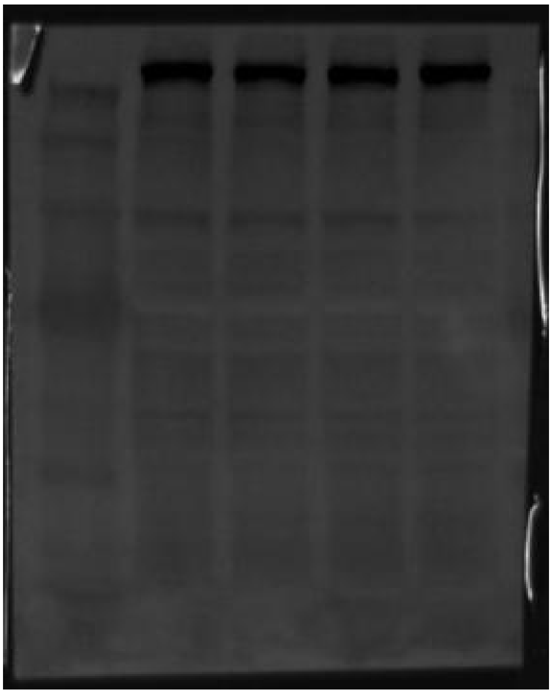

Fig8A:mTOR-GAPDH 36kDa

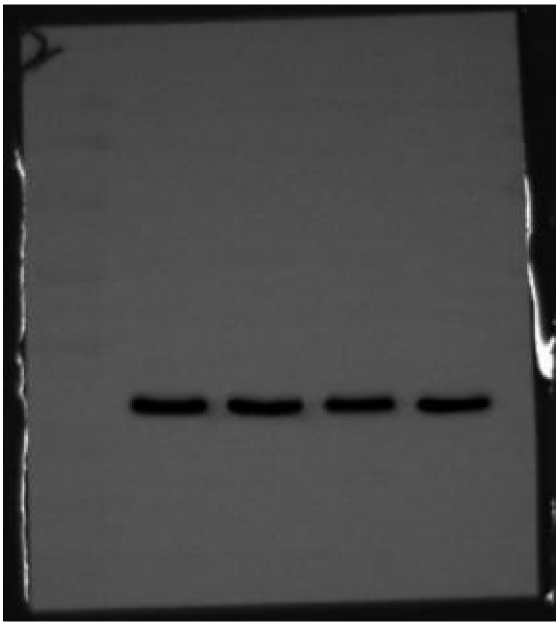

Supplement: Supplementary file 3 — Figure S1: jcmm71128‐sup‐0003‐FigureS1.pdf. [file JCMM-30-e71128-s003.pdf]
